# The Histone Demethylase Enzymes KDM3A and KDM4B Co-Operatively Regulate Chromatin Transactions of the Estrogen Receptor in Breast Cancer

**DOI:** 10.3390/cancers11081122

**Published:** 2019-08-06

**Authors:** Dominic Jones, Laura Wilson, Huw Thomas, Luke Gaughan, Mark A. Wade

**Affiliations:** 1 Northern Institute for Cancer Research, Newcastle University, Newcastle upon Tyne NE2 4HH, UK; 2 Biomedical Sciences, Faculty of Health Sciences, University of Hull, Hull HU6 7RX, UK

**Keywords:** epigenetics, histone demethylation, estrogen receptor, breast cancer

## Abstract

Many estrogen receptor (ER)-positive breast cancers develop resistance to endocrine therapy but retain canonical receptor signalling in the presence of selective ER antagonists. Numerous co-regulatory proteins, including enzymes that modulate the chromatin environment, control the transcriptional activity of the ER. Targeting ER co-regulators has therefore been proposed as a novel therapeutic approach. By assessing DNA-binding dynamics in ER-positive breast cancer cells, we have identified that the histone H3 lysine 9 demethylase enzymes, KDM3A and KDM4B, co-operate to regulate ER activity via an auto-regulatory loop that facilitates the recruitment of each co-activating enzyme to chromatin. We also provide evidence that suggests that KDM3A primes chromatin for deposition of the ER pioneer factor FOXA1 and recruitment of the ER-transcriptional complex, all prior to ER recruitment, therefore establishing an important mechanism of chromatin regulation involving histone demethylases and pioneer factors, which controls ER functionality. Importantly, we show via global gene-expression analysis that a KDM3A/KDM4B/FOXA1 co-regulated gene signature is enriched for pro-proliferative and ER-target gene sets, suggesting that abrogation of this network could be an efficacious therapeutic strategy. Finally, we show that depletion of both KDM3A and KDM4B has a greater inhibitory effect on ER activity and cell growth than knockdown of each individual enzyme, suggesting that targeting both enzymes represents a potentially efficacious therapeutic option for ER-driven breast cancer.

## 1. Introduction

Estrogen receptor (ER) signalling is important for breast cell homeostasis and transformation and remains the primary target for therapeutic intervention in breast cancer (BC). Typically, ER-mediated transactivation is under tight control by a myriad of co-factors regulating the transcriptional competency of the receptor at numerous levels [1,2]. Several lines of evidence have indicated that chromatin deposition of pioneer factors FOXA1 and GATA3 at ER-target loci is critical for enabling appropriate DNA interactions of the receptor [3,4]. Subsequent recruitment of enzymes that modulate the chromatin environment, such as the histone acetyltransferase (HAT) p300, further stimulates ER signalling by enhancing recruitment of the RNA polymerase machinery [1,5]. Additional down-stream trans-acting factors, such as the bromodomain and extra-terminal (BET) protein BRD4, which promotes the elongation stage of mRNA synthesis, are also vital for ER-induced gene expression [6].

Critically, aberrant activity of ER co-regulators drives the progression of BC [1,2,7,8,9]. In light of the fact that therapeutically targeting the ER directly in BC routinely fails, developing treatments that target critical ER co-regulators may provide an effective second-line treatment for endocrine therapy-resistant disease. Understanding how these co-factors work and interact to regulate ER signalling is therefore crucial.

Dynamic and selective methylation of lysines on histones H3 and H4 contributes a critical layer of epigenetic regulation to control gene transcription [10,11]. In contrast to lysine methylation of histone H3 lysine 4 (H3K4me) and 36 (H3K36me), which is principally associated with transcriptional activation, modification of histone H3 lysine 9 (H3K9me) and 27 (H3K27me) is enriched at transcriptionally inert regions of the genome. Additional complexity is provided by the existence of three isomeric states of methylation where target lysines can be either mono-, di-, or tri-methylated (me1, me2, me3). Histone methylation is catalysed by the Su(var)3-9, Enhancer-of-zeste, Trithorax (SET) domain family of histone lysine methyltransferases (KMTs) and is actively removed by histone lysine demethylase (KDM) enzymes, of which there are eight characterised families. All but one KDM family (KDM1) contain the catalytically active Jumanji-C (JmjC) demethylase domain [11,12,13,14]. Aberrant expression and activity of KMTs and KDMs and aberrant histone methylation patterns have been associated with cancer [15,16,17,18].

Two KDM enzymes, KDM3A and KDM4B, which remove transcriptionally repressive H3K9me1/me2 and H3K9me2/me3 marks, respectively, have both been associated with oncogenic roles in numerous cancer types, suggesting that perturbation of their activity may be efficacious as a therapeutic option [19,20,21,22,23,24,25,26,27,28,29,30,31,32,33,34]. Interestingly, KDM3A and KDM4B have both been identified as key regulators of a number of transcription factors, including androgen receptor (AR), p53, and HIF1α [22,30,32,35,36,37,38,39], and both play roles in embryonic stem cell development and metabolic homeostasis [40,41,42,43,44,45,46]. Furthermore, our previous findings have shown that both enzymes facilitate ER-mediated transcription by erasure of methylated H3K9 marks and promotion of ER chromatin binding at receptor target genes [20,47]. These observations suggest that KDM3A and KDM4B co-operate in their regulation of oncogenic signalling pathways. However, functional interplay between the two enzymes has not yet been investigated, particularly in the context of ER signalling.

Here we show that KDM3A and KDM4B interact in BC cells and are involved in an auto-regulatory loop whereby KDM4B regulates the expression of KDM3A, and KDM3A facilitates the recruitment of KDM4B to ER *cis*-regulatory elements. Subsequently, depletion of both KDM3A and KDM4B has a greater inhibitory effect on receptor activity than depletion of individual enzymes. We also provide evidence that KDM3A is required for chromatin occupancy of FOXA1, suggesting that the demethylase acts as a key regulator of pioneer factor-chromatin interactions and provides an appropriate environment for subsequent recruitment of trans-acting factors, such as BET proteins and p300. FOXA1 expression is also regulated by KDM4B, and we show that the transcriptomes of KDM3A, KDM4B, and FOXA1 overlap considerably. Gene set enrichment analysis indicates that co-regulated genes are involved in ER signalling and cell cycle regulation, suggesting an important role for this regulatory network in driving ER-positive BC growth. In support of this, we demonstrate that depletion of KDM3A and KDM4B together has a greater inhibitory effect on cell proliferation than the depletion of each enzyme on its own. Together, our findings suggest a co-regulatory relationship between KDM3A and KDM4B on ER-signalling via FOXA1 regulation and deposition, and that targeting both KDM enzymes would be an efficacious therapeutic option for ER-positive and endocrine therapy-resistant disease.

## 2. Results

### 2.1. KDM3A Interacts with KDM4B and Regulates Chromatin Occupancy in ER-Positive Breast Cancer 

KDM3A and KDM4B both play roles in similar oncogenic signalling pathways [20,22,30,32,35,36,37,38,39,47]. However, a co-operative role between the two enzymes has not been studied. We have previously shown that both KDM3A and KDM4B are required for ER recruitment to *cis*-regulatory elements of ER target genes [20,47]. The knockdown of KDM3A and KDM4B individually abrogated the recruitment of the ER to oestrogen response elements (EREs) following oestrogen (E_2_) stimulation and abrogated the demethylation of target H3K9 methylation marks (KDM3A H3K9me1/2; KDM4B H3K9me3). We therefore investigated interactions between KDM3A and KDM4B in ER-positive BC.

**Figure 1:**
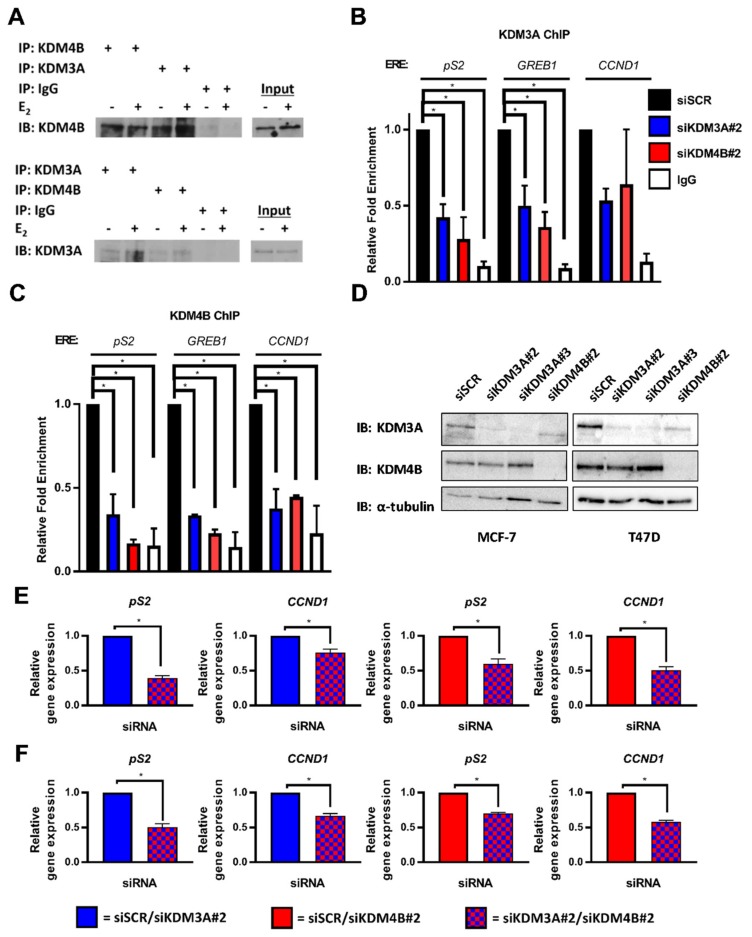
KDM3A interacts with KDM4B and regulates chromatin occupancy. (**A**) MCF-7 cells grown in steroid-depleted (−E_2_) and 10 nM E_2_-supplemented (+E_2_) media were subject to immunoprecipitation (IP) using anti-KDM4B, anti-KDM3A, or isotype control (IgG) antibodies before western blot analysis (IB) using reciprocal antibodies. (**B**,**C**) MCF-7 cells were transiently transfected with either siSCR, siKDM3A#2, or siKDM4B#2 and grown in steroid-depleted conditions for 72 h prior to treatment with 10 nM E_2_ for 45 min followed by ChIP analysis using antibodies specific to KDM3A (**B**) or KDM4B (**C**) and isotype controls (IgG). ChIP using the isotype control antibody was performed on siSCR transfected cells in each experiment. Enrichment of KDM3A and KDM4B at *pS2*, *GREB1*, and *CCND1* oestrogen response elements (EREs) was assessed by qPCR. Data are an average of 2 independent experiments ± SEM and are expressed relative to the level of enrichment measured in the siSCR transfected cells. *P*-values were determined by Dunnet’s multiple comparisons test (* denotes *p* < 0.05). (**D**) MCF-7 and T47D cells were transiently transfected with either siSCR, siKDM3A#2, siKDM3A#3, or siKDM4B#2 and grown in steroid-depleted conditions for 72 h prior to treatment with 10 nM E_2_ for 4 h and then western blot analysis using antibodies specific to KDM4B, KDM3A, and α-tubulin. α-tubulin was used to compare protein loading between samples. (**E**,**F**) *pS2* and *CCND1* gene expression in MCF-7 (**E**) or T47D (**F**) cells transfected with either an siRNA mixture of siSCR and siKDM3A#2 (red) or siSCR and siKDM4B#2 (blue) (single knockdowns) and compared with expression in cells transfected with an siRNA cocktail of siKDM3A#2 and siKDM4B#2 (red and blue) (dual knockdown). Cells were transfected and grown in steroid depleted media for 72 h prior to stimulation with E_2_ for 4 h and RNA extraction. qPCR data are an average of 3 repeats ± SEM and are expressed relative to gene expression in single gene knockdown cells. *P* values were determined by Students *T* test (* = *p* < 0.05).

By probing endogenous KDM3A and KDM4B immunoprecipitates with reciprocal antibodies, we identified that KDM3A and KDM4B interact in MCF-7 (Figure 1A) and T47D (Supplementary Figure S1A) cells in both 10 nM E_2_-stimulated (+E_2_) and steroid-depleted (−E_2_) growth conditions. Given that our previous studies have shown that both KDM3A and KDM4B are enriched at *cis*-regulatory elements of ER-target genes [20,47], we investigated whether the demethylases are co-dependent upon each other for chromatin binding. Using chromatin immunoprecipitation (ChIP) in MCF-7 cells, we identified that KDM3A and KDM4B occupied *cis*-regulatory elements of *pS2*, *GREB1,* and *CCND1* genes in the presence of E_2_ (Figure 1B,C). Importantly, chromatin binding of KDM3A was attenuated upon depletion of KDM4B and, reciprocally, KDM4B occupancy was diminished by the knockdown of KDM3A, suggesting that the demethylases work co-operatively to facilitate their chromatin association. Interestingly, through the course of these experiments, we observed that KDM4B depletion downregulated KDM3A mRNA and protein expression in MCF-7 cells, and KDM3A protein expression in T47D cells (Figure 1D; Supplementary Figure S1B). KDM4B depletion did not downregulate KDM3A mRNA expression, suggesting that the mechanisms of regulation, or temporal dynamics of this regulation, differs between the cell lines. The effect of KDM4B depletion on KDM3A expression likely explains reduced chromatin occupancy of KDM3A observed in our ChIP experiments. Critically, no effect on KDM4B protein expression was observed following KDM3A knockdown in either MCF-7 or T47D cells (Figure 1D), therefore suggesting that KDM3A specifically regulates the recruitment of KDM4B to ER target genes and that an auto-regulatory loop exists between the two enzymes.

Considering the proposed co-operative role of KDM3A and KDM4B in ER-signalling, we next assessed the expression of the ER-target genes *pS2* and *CCND1* in dual KDM3A- and KDM4B-depleted cells compared to the knockdown of individual enzymes. Consistent with our previous findings, depletion of both KDM3A and KDM4B down-regulated the expression of *pS2* and *CCND1* (Supplementary Figure S2A,B). Importantly, expression of the ER-target genes in both MCF-7 and T47D cells was significantly lower in dual KDM3A- and KDM4B-depleted cells compared to the knockdown of individual enzymes (Figure 1E,F; Supplementary Figure S3), suggesting that interplay between KDM3A and KDM4B is required for ER-mediated transactivation. The effect of dual KDM3A- and KDM4B- depletion on respective KDM protein expression in MCF-7 and T47D cells can be seen in Supplementary Figure S3A.

### 2.2. KDM3A Facilitates the Pioneering Role of FOXA1 in ER-Positive Breast Cancer

**Figure 2:**
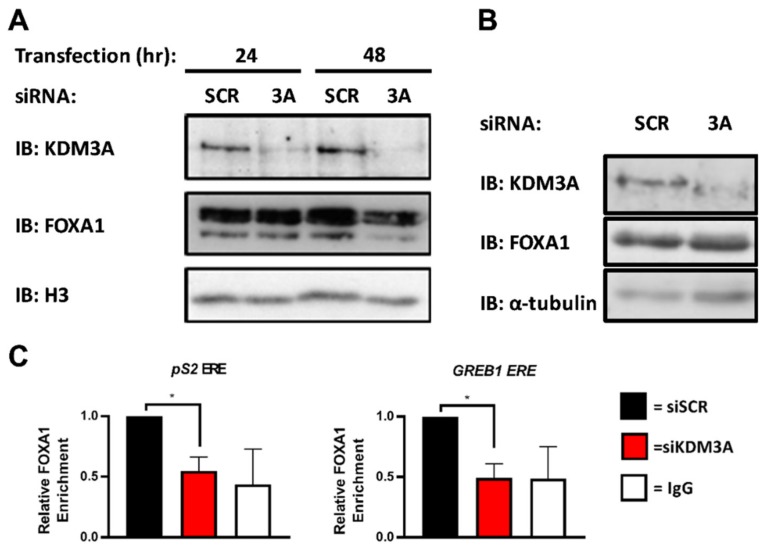
KDM3A is required for FOXA1 recruitment to EREs. (**A**) MCF-7 cells were transiently transfected with either siSCR or siKDM3A#1. Cells were harvested after 24 and 48 h and cell fractionation performed prior to western blot analysis of proteins from the chromatin fraction using antibodies specific to KDM3A, FOXA1, and histone H3 as a loading control. (**B**) MCF-7 cells were transiently transfected with either siSCR or siKDM3A#1 and grown for 48 h prior to western blot analysis using antibodies specific to KDM3A, FOXA1, and α-tubulin. (**C**) MCF-7 cells were transiently transfected with either siSCR or siKDM3A#1 and grown in steroid-depleted conditions for 48 h prior to treatment with 10 nM E_2_ for 45 min followed by ChIP analysis using antibodies specific to FOXA1 and isotype controls (IgG). ChIP using the isotype control antibody was performed on siSCR transfected cells in each experiment. Enrichment of FOXA1 at a *pS2* proximal promoter ERE and *GREB1* enhancer ERE was assessed by qPCR. Data are an average of 3 independent experiments ± SEM and are expressed relative to the level of enrichment measured in siSCR transfected cells. *p*-values were determined by Student’s *t*-test (* denotes *p* < 0.05).

Previous studies have demonstrated that chromatin association of KDM3A, but not KDM4B, precedes ER recruitment [20,47]. KDM3A was present at EREs in −E_2_ conditions, whereas KDM4B was actively recruited to EREs following E_2_ stimulation. Given that KDM3A chromatin occupancy is evident at quiescent ER-target genes, we speculated that KDM3A dependent recruitment of KDM4B may be due to chromatin regulation prior to ER activation. The pioneer factor FOXA1 plays a pivotal role in regulating chromatin occupancy and transcriptional competency of the ER in BC, with >50% of the ER cistrome overlapping with sites of FOXA1 deposition and approximately 95% of ER-target genes requiring FOXA1 for estrogenic activation, respectively [3]. What controls FOXA1 chromatin binding is still unclear, however evidence suggests that the presence of the transcriptionally permissive histone H3 lysine 4 methylation (H3K4me) mark and absence of transcriptionally repressive H3K9me marks are key determinants [48]. KDM3A knockdown was previously shown to increase H3K9 di-methylation at quiescent ER-target gene cis-regulatory sites [20]. We therefore speculated that KDM3A might be a regulator of FOXA1 DNA binding. To this end, BC cells depleted of KDM3A for 24 and 48 h were subject to chromatin extraction prior to western analysis using anti-FOXA1 and anti-KDM3A antibodies. Knockdown of KDM3A reduced FOXA1 chromatin association in both MCF-7 and T47D cell lines (Figure 2A; Supplementary Figure S4A), which was not a consequence of reduced total FOXA1 protein or mRNA levels (Figure 2B; Supplementary Figure S4B,C). More specifically, KDM3A depletion reduced FOXA1 enrichment at promoter and enhancer elements of ER-target genes *pS2* and *GREB1* in MCF-7 cells by ChIP (Figure 2C; Supplementary Figure S4D). These results therefore suggest that KDM3A may act upstream of ER-recruitment in the ER signalling pathway and regulate FOXA1 deposition at these loci, therefore highlighting a potential mechanism of chromatin regulation prior to ER activation which in turn could regulate KDM4B recruitment to ER-target genes and the construction of the ER transcriptional complex.

To further support this finding, we assessed if KDM3A controlled the chromatin association of other co-regulators associated with ER-mediated transactivation in BC cells. The binding of the acetyl transferase p300 and the bromodomain and extra-terminal (BET) protein BRD4 (both downstream co-regulators of ER signalling) was assessed by ChIP in MCF-7 cells depleted of KDM3A in the presence and absence of 10 nM E_2_. Consistent with activation of ER signalling, E_2_ treatment enhanced both p300 and BRD4 recruitment to regulatory elements of *pS2* and *GREB1* genes, but not a control region in the *pS2* promoter, which does not bind ER (Supplementary Figure S5A). As expected, upon depletion of KDM3A, E_2_-stimulated recruitment of p300 and BRD4 at these loci was markedly reduced, which concurrently down-regulated the acetylation of histone H3 lysine 27 (H3K27ac) (Supplementary Figure S5A). Importantly, global p300 and BRD4 levels were not impacted by KDM3A knockdown (Supplementary Figure S5B), confirming the role of KDM3A in the construction of the ER transcriptional complex and demonstrating that KDM3A-driven H3K9me2 demethylation also induces histone acetylation.

### 2.3. KDM3A, KDM4B, and FOXA1 Have Overlapping Pro-Proliferative Gene Regulatory Profiles in ER Positive Breast Cancer

**Figure 3:**
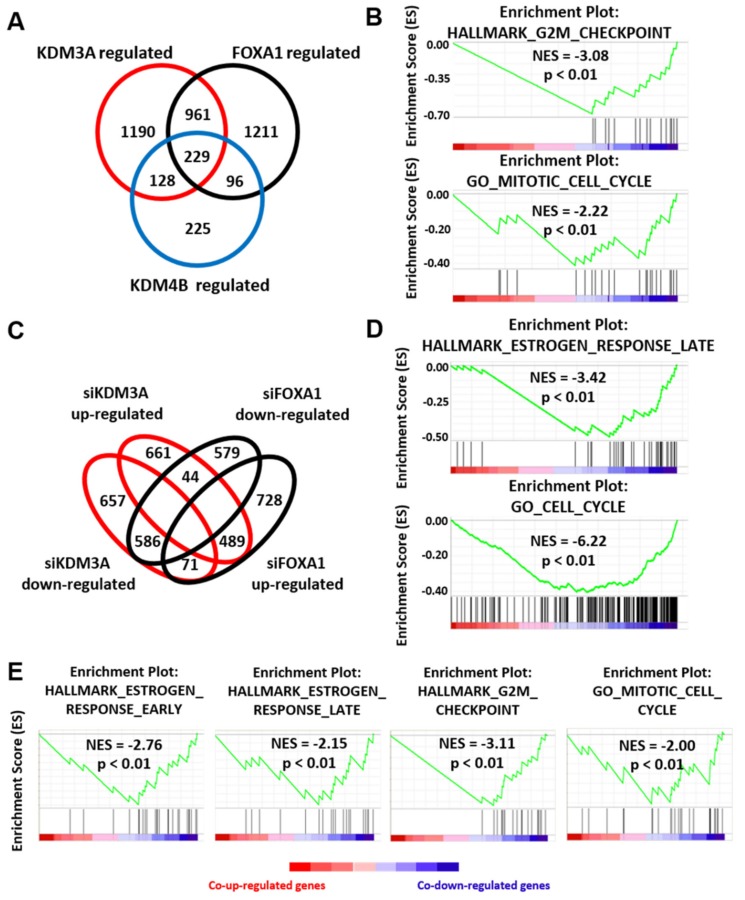
KDM3A, KDM4B, and FOXA1 have overlapping gene regulatory profiles. (**A**) MCF-7 cells were transiently transfected with either a non-silencing control siRNA (siSCR), siKDM3A#1, siKDM4B#1, or siFOXA1#1 and grown in steroid-depleted medium for 48 h prior to treatment with 10 nM E_2_. RNA was extracted and samples hybridized to an Illumina HT12.2 Bead CHIP array. The number of KDM3A- (*n* = 2508), KDM4B- (*n* = 678), and FOXA1-regulated (*n* = 2497) genes (defined as genes significantly (*p* < 0.05) up or down regulated 1.5 fold upon knockdown compared to siSCR controls) was determined. Genes up- or down-regulated by more than one enzyme were classified as KDM3A/KDM4B/FOXA1 co-regulated genes (*n* = 1190 KDM3A/FOXA1 co-regulated genes; *n* = 357 KDM3A/KDM4B co-regulated genes; 325 KDM4B/FOXA1 co-regulated genes; *n* = 229 KDM3A/KDM4B/FOXA1 co-regulated genes). (**B**) GSEA of KDM3A/KDM4B/FOXA1 co-regulated genes demonstrates significant negative enrichment of genes associated with the HALLMARK_G2M_CHECKPOINT geneset and GO_MITOTIC_CELL_CYCLE geneset. NES = Nomalised Enrichment Score. *p* = nominal *p*-value. (**C**) Comparison between KDM3A- and FOXA1- up- and down-regulated genesets. (**D**) GSEA of KDM3A/FOXA1 co-regulated genes demonstrates significant negative enrichment of genes associated with the HALLMARK_ESTROGEN_RESPONSE_LATE geneset and GO_CELL_CYCLE geneset. (**E**) GSEA of KDM3A/KDM4B co-regulated genes demonstrates significant negative enrichment of genes associated with the HALLMARK_ESTROGEN_RESPONSE_EARLY, HALLMARK_ESTROGEN_RESPONSE_LATE and HALLMARK_G2M_CHECKPOINT genesets and the GO_MITOTIC_CELL_CYCLE geneset.

KDM4B has previously been shown to regulate FOXA1 expression [47]. Coupled with the finding that KDM4B also regulates KDM3A expression and that KDM3A regulates both KDM4B and FOXA1 deposition at ER *cis*-regulatory sites, our data suggests a potential co-regulatory relationship between all three proteins to facilitate a cancer phenotype in ER-positive BC. To investigate this further, we examined the overlap between KDM3A-, KDM4B-, and FOXA1-regulated transcriptomes in MCF-7 cells by microarray analysis. Using a 1.5-fold cut-off for differentially-expressed genes between control and enzyme-depleted cells, we identified 229 genes that were co-regulated by all three enzymes, of which 213 (93%) were differentially expressed in the same direction (Figure 3A). Gene set enrichment analysis (GSEA) identified the enrichment of co-down-regulated genes in cell cycle processes, particularly genes that were involved in controlling the G2/M checkpoint, indicating a shared regulatory function in ER-positive BC growth (Figure 3B; Supplementary Table S1). 

Significant overlap was observed between KDM3A and FOXA1 gene regulatory profiles, with 43% of KDM3A-regulated genes co-regulated by FOXA1 and 43% of FOXA1-regulated genes also regulated by KDM3A. Of 1075 KDM3A and FOXA1 co-regulated genes, 586 were down-regulated and 489 were up-regulated, with only 115 co-regulated genes regulated in opposing directions (Figure 3C), indicating shared transcriptomic profiles between KDM3A and FOXA1. Importantly, KDM3A knockdown had no impact on FOXA1 levels (Figure 2B) and, likewise, FOXA1 depletion by two independent siRNAs (siFOXA1#1 and siFOXA1#2) had no effect on KDM3A expression (Supplementary Figures S4C and Figure S6A), confirming that the effects of KDM3A and FOXA1 knockdown is a consequence of altered transcriptional activity. As expected, GSEA of co-down-regulated genes highlighted early and late estrogen-stimulated gene signatures, indicating a co-regulatory role in ER-mediated gene transcription, but also identified other oncogenic signatures, including E2F and MYC (Figure 3D; Supplementary Table S2; Supplementary Figure S6B). GSEA also identified that a significant number of co-down-regulated genes are required for cell cycle processes (Figure 3D; Supplementary Table S2). Similarly, genes up-regulated in response to KDM3A and FOXA1 depletion were associated with growth inhibition and p53 pathway gene signatures (Supplementary Table S3; Supplementary Figure S6B).

Interestingly, we identified that 53% and 48% of KDM4B-regulated genes were also regulated by KDM3A and FOXA1, respectively. Of 357 KDM4B and KDM3A co-regulated genes, 336 were differentially expressed in the same direction (168 down-regulated and 168 up-regulated), while out of 325 KDM4B and FOXA1 co-regulated genes, 282 were differentially expressed in the same direction (141 down-regulated and 141 up-regulated). Further, 63% of KDM4B and KDM3A co-regulated genes were also regulated by FOXA1 and 75% of KDM4B and FOXA1 co-regulated genes were also regulated by KDM3A, showing the intrinsic link between KDM4B and KDM3A/FOXA1 regulated gene signatures. As expected, KDM4B and FOXA1 co-down-regulated genes were associated with early estrogen response gene signatures and cell cycle processes (Supplementary Table S4). Consistent with our previous findings [20,47], genes down-regulated by depletion of both KDM3A and KDM4B were also associated with early and late estrogen response gene signatures (Figure 3E; Supplementary Table S5). KDM3A and KDM4B co-down-regulated genes were also involved in cell cycle regulation, suggesting that KDM3A and KDM4B co-operate in driving a pro-proliferative phenotype (Figure 3E; Supplementary Table S5).

Interestingly, the analysis of genes regulated independently by either KDM3A (*n* = 1190), KDM4B (*n* = 225), or FOXA1 (*n* = 1211) were not enriched for the cell cycle or cell proliferation processes. This suggests that the co-regulatory relationship between KDM3A/KDM4B/FOXA1 is key to ER-positive BC-growth and that abrogation of this regulatory axis could be a promising therapeutic approach.

### 2.4. Dual Targetting of KDM3A and KDM4B is Potentially an Efficacious Therapeutic Strategy

Considering that global transcriptomic analysis suggested that KDM3A and KDM4B co-operate to drive a pro-proliferative phenotype and that dual knockdown of each enzyme down-regulated ER-target genes more than individual KDM3A and KDM4B depletion, we next assessed the effect of dual-knockdown on BC cell growth.

Previous work has shown that KDM4B is required for ER-positive BC growth both in vitro and in vivo, demonstrating its potential as a novel therapeutic target [28,29]. We have also previously shown that KDM3A depletion inhibits BC cell growth in vitro, demonstrating its therapeutic potential [20]. In a small mouse xenograft pilot study using a derivative MCF-7 cell line that stably expresses an inducible KDM3A-targeting shRNA under the control of doxycycline, we also provide proof-of-principle analysis that KDM3A depletion inhibits in vivo BC growth (Supplementary Figures S7 and S8). Although mouse numbers were small and therefore it is difficult to make conclusions from this analysis, data from this pilot study is consistent with our previous in vitro findings and suggests that KDM3A is a driver of BC cell proliferation.

**Figure 4:**
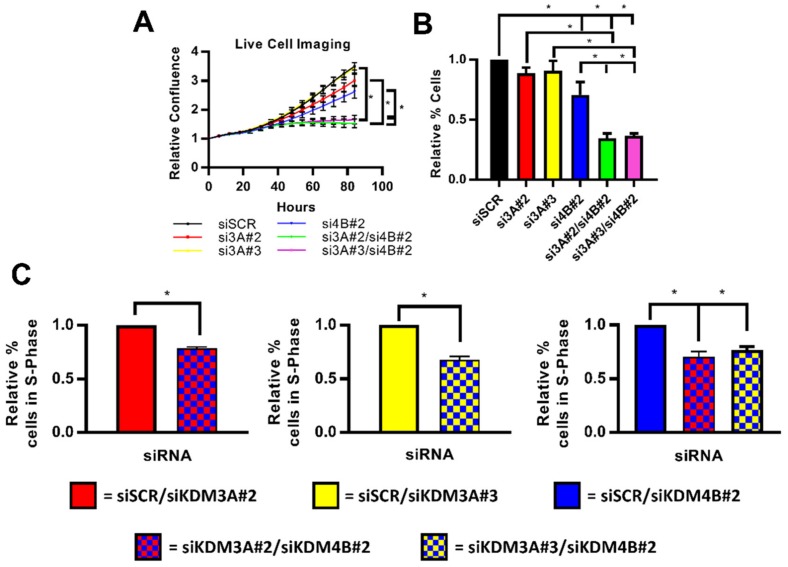
Inhibition of cell growth is enhanced following dual knockdown of KDM3A and KDM4B. (**A**–**C**) T47D cells were grown in steroid-depleted conditions for 48 h and were then treated with 10 nM E_2_ for 24 h before transfection with either siSCR or single and dual-knockdown siRNA mixtures (see materials and methods). Data labels indicate which KDM siRNAs were included in each siRNA mixture. (**A**) Cell confluence was measured every 6 h post-transfection by the Incucyte Zoom live cell imager. Data was normalised for each experimental arm to the cell confluence measured at 0 h. Data are the average of three independent experiments ± SEM. *P*-values were determined by Turkey’s multiple comparisons test (* denotes *p* < 0.05). (**B**) Cell counts were taken 96 h post transfection. Data are the average of three independent experiments ± SEM and are expressed relative to cell counts for siSCR transfected cells. *P*-values were determined by Turkey’s multiple comparisons test (* denotes *p* < 0.05). (**C**) Cells were harvested and stained with propidium iodide 96 h post transfection for flow cytometry analysis. Data shows the comparison of the percentage of cells in S-phase of the cell cycle between cells transfected with a single-knockdown siRNA mixture and a dual-knockdown siRNA mixture. Data labels indicate the siRNAs used in each mixture. Data are an average of 3 repeats ± SEM and are expressed relative to the percentage of S-phase cells in single gene knockdown cells. *P* values were determined by Students *T* test (* = *p* < 0.05).

To test the effect of dual knockdown on cell growth, we compared the growth of both T47D and MCF-7 cells depleted of individual or both demethylases in E_2_-containing conditions after 96 h. In both cell lines, inhibition of cell growth was enhanced following dual knockdown compared to depletion of either enzyme on its own (Figure 4A,B (T47D); Supplementary Figure S9 (MCF-7)). Moreover, cell cycle analysis in T47D cells showed a decrease in cells in S-phase following dual-knockdown, confirming enhanced inhibition of cell proliferation (Figure 4C). Considering the proposed co-regulatory relationship between KDM3A, KDM4B, and FOXA1, which facilitates a cancer phenotype in ER-positive BC, these data together suggest that targeting KDM3A and KDM4B simultaneously may be an efficacious therapeutic strategy.

## 3. Discussion

Estrogen receptor signalling involves a myriad of pioneer and co-regulatory proteins, demonstrating the complexity of transcriptional regulation of the receptor [1,2]. Approximately two thirds of BCs rely on ER-mediated transcriptional activation for growth. Endocrine therapy, which targets the interaction between the ER and estrogen, is currently the main therapeutic option. However, many women develop resistance to ER-targeting agents. The majority of resistant tumours maintain ER-signalling via numerous mechanisms including changes in activity of co-regulatory proteins and increased ER activation by kinase signalling pathways [49]. Targeting alternative proteins within the ER-signalling cascade is therefore emerging as a promising therapeutic option in both ER-positive and endocrine therapy-resistant disease.

**Figure 5:**
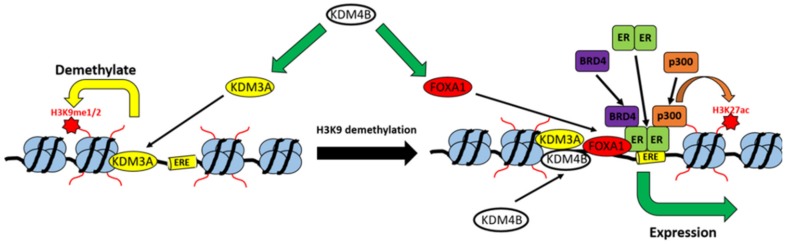
Model of the KDM3A/KDM4B co-regulatory network. KDM4B is required for the expression of both KDM3A and FOXA1. KDM3A demethylates H3K9me1/me2 marks at EREs, which is required for the recruitment of both KDM4B and FOXA1, which in-turn is required for the recruitment of the ER-transcriptional complex, associated trans-activating chromatin modifications, and the expression of ER-target genes.

We have previously identified KDM3A and KDM4B as co-activators of ER-activity [20,47]. In this study, we identified that KDM3A and KDM4B interact in BC cells and are involved in an auto-regulatory loop in which KDM4B regulates KDM3A expression and KDM3A facilitates the recruitment of KDM4B to EREs. KDM4B is also a regulator of expression of the ER pioneer protein FOXA1, and KDM3A appears to be required for the deposition of FOXA1 on chromatin and the subsequent recruitment of ER co-activator proteins and deposition of transcriptionally activating chromatin modifications, therefore highlighting a key co-regulatory role that these two KDM enzymes have on ER signalling (represented in Figure 5). Gene expression analysis following the knockdown of each of these proteins identified a KDM3A/KDM4B/FOXA1 co-regulatory transcriptomic profile enriched for genes involved in cell cycle regulation, suggesting that this is a key pro-proliferative regulatory network in ER-positive BC cells. Further global cistromic analysis of the effect of knockdown of KDM3A and KDM4B on the recruitment and deposition of each enzyme and of FOXA1 would further elucidate the interplay between these three proteins.

FOXA1 is a key ER pioneer factor and is required for the transactivation of a large proportion of the canonical ER [3]. Considering the apparent co-regulatory role that KDM3A and KDM4B have on FOXA1 deposition, dual blockade of the enzymes may be an effective strategy to comprehensively abrogate the FOXA1/ER network. We identified that depletion of both KDM3A and KDM4B together has a greater inhibitory effect on both ER-target gene expression and BC cell growth than targeting either enzyme individually, suggesting that dual targeting of KDM3A and KDM4B may have significant clinical applications in ER-positive BC. The development of both pan-KDM and semi-selective KDM family inhibitors have shown promise as anti-cancer therapeutic agents [50,51]. For example, semi-selective KDM4 inhibitors that abrogate KDM4B activity have been shown to be efficacious in inhibiting AR-signalling and growth in prostate cancer cells and to be effective in inhibiting the growth of MYC-driven neuroblastomas and breast cancer stem-like cells [52,53,54,55]. The conserved protein structure between JmjC-domain containing KDM families makes it challenging to develop potent single-enzyme selective inhibitors. However, the identification of a co-regulatory role between enzymes from different KDM families may suggest that a less selective approach could still be efficacious as a therapeutic strategy.

Recent findings have suggested roles for both KDM3A and KDM4B in enhancing ER-signalling in endocrine-therapy resistant BC [56,57]. Considering the co-regulatory role of KDM3A and KDM4B in ER-signalling, dual targeting of these enzymes may provide new therapeutic strategies to address the unmet clinical need for treatments of this disease stage. In many endocrine therapy-resistant breast cancers, the antagonist-bound ER cistrome is distinct from that of the estrogen-activated ER, thereby creating an alternative ER-transcriptome that enables disease progression [58]. FOXA1 is overexpressed in endocrine therapy-resistant disease and mediates the alteration of ER-chromatin interactions [3,58,59]. Whether KDM3A plays a role in FOXA1 and ER deposition in endocrine therapy-resistant cells is currently unknown, although our previous data suggested that KDM3A abrogates ER-signalling in a model of tamoxifen resistance [20]. Phosphorylation of KDM3A has also been shown to promote ER binding to the promoter of the oncogene *HOXA1* in tamoxifen treated BC cells [57], suggesting that KDM3A plays a role in regulating ER recruitment in endocrine therapy-resistant cells. Whether this effect is mediated by FOXA1 binding, however, is currently unknown. 

An alternative mechanism of endocrine-therapy resistance includes cross-talk between estrogen and hypoxia-mediated signalling pathways [60]. HIF-1α expression is associated with resistance to the ER antagonist tamoxifen and overactive HIF-1α is suggested to compensate for abrogated ER-signalling in endocrine therapy-resistant disease [60,61]. The hypoxia inducible transcription factor, *HIF-1α*, is itself an ER-regulated gene and HIF-1α and ER have common gene targets [62]. *KDM4B* is a hypoxia- and estrogen-induced gene and has been proposed to be a key link between the two signalling pathways [63]. KDM3A is also regulated by HIF-1α and is a key modulator of HIF-1α signalling [37,38]. Considering the co-regulatory role between KDM3A and KDM4B in ER signalling, this suggests that both of these enzymes may be key modulators between the estrogen and hypoxia pathways. Inhibition of both enzymes may therefore be effective in treating hypoxia-induced endocrine therapy resistance. Hypoxia is also an independent oncogenic driver and both KDM3A and KDM4B are co-activators of HIF-1α signalling in multiple cancer types [33,37,38,64]. The study of a co-regulatory mechanism between KDM3A and KDM4B in hypoxic signalling may therefore also be beneficial for the development of novel therapeutic strategies to abrogate hypoxia driven tumours. 

As discussed, KDM3A and KDM4B have both been identified as key regulators of multiple transcription-factors [22,30,32,35,36,37,38,39]. To the best of our knowledge, this is the first study to demonstrate a direct co-regulatory link between KDM3A and KDM4B in a shared signalling pathway. It is possible that similar co-regulatory functions exist in other oncogenic signalling pathways. For example, both KDM3A and KDM4B have been shown to attenuate the expression of p53-regulated genes [35,36]. Methylation of K372 on p53 activates the recruitment of the transcription factor to target gene promoters. KDM3A demethylates K372 on p53 and therefore inhibits p53 binding and transcriptional activity [35]. The depletion of KDM3A was shown to reactivate mutant p53 activity in breast cancer. The mechanism by which KDM4B attenuates p53-regulated gene activation has not been identified, however it was hypothesised that KDM4B upregulates a p53 transcriptional repressor [36]. The findings of our study may therefore provide a mechanistic link between KDM4B and p53 attenuation via the upregulation of KDM3A, which warrants further investigation. 

Overall, the identification of a KDM3A/KDM4B co-regulatory axis that regulates FOXA1 and subsequent ER deposition on chromatin in breast cancer enhances the understanding of the mechanisms controlling ER-transcriptional activity. Crucially, the finding that dual KDM3A and KDM4B depletion has an additive anti-proliferative effect identifies a novel therapeutic approach for both ER-positive and endocrine therapy-resistant disease. 

## 4. Materials and Methods 

### 4.1. Cell Culture

MCF-7, T47D, and HEK293T cells (ATCC) were maintained in RPMI-1640 media (Sigma, Saint Louis, MO, USA) containing 10% foetal-calf serum (FCS) (Gibco, Paisley, UK) and 1% penicillin/streptomycin (Sigma). For steroid-depletion experiments, cells were grown in phenol red-free RPMI-1640 media (Gibco) supplemented with 10% serum stripped FCS (Hyclone, South Logan, UT, USA) and 1% penicillin/streptomycin. For estrogen stimulation experiments, 10 nM 17-β-estradiol (E_2_) (Sigma) was added for the defined stimulation period.

### 4.2. siRNA Transfection

For knockdown experiments, three KDM3A-, two KDM4B-, two FOXA1-targeting, and control (siSCR) siRNAs were used (Sigma) (Supplementary Table S6). Cell lines were transfected using Lipofectamine RNAiMAX (Invitrogen, Carlsbad, CA, USA) with siRNA mixtures to a final concentration of 25 nM, as previously described [65]. For dual knockdown experiments, siRNA mixtures of two individual siRNAs were made to a final concentration of 25 nM (12.5 nM per siRNA). To assess the expression of KDM3A, KDM4B, FOXA1, p300, BRD4, and α-tubulin, transfected cells were harvested in SDS-sample buffer (10% β-mercaptoethanol, 125 mM Tris-HCl (pH 6.8), 2% SDS, 10% glycerol, and 0.005% bromophenol blue) and were subject to polyacrylamide gel electrophoresis prior to immunoblotting with specific antibodies as described in [66] (Supplementary Table S7). For the chromatin extraction experiments, transfected cells underwent cell fractionation using the NE-PER Nuclear and Cytoplasmic Extraction kit (Thermo Scientific, Waltham, MA, USA). Cell fractions were harvested in SDS-sample buffer and probed for KDM3A, FOXA1, and histone H3 by western blot. For gene expression analysis, RNA was extracted from transfected cells using TRIzol (Ambion, Austin, TX, USA) and cDNA generated for analysis by quantitative PCR (qPCR) [65] (see Supplementary Table S8 for primer sequences).

### 4.3. Microarray and Gene Set Enrichment Analysis

MCF-7 cells were transfected with either siSCR or siKDM4B#1, as described above, and grown for 48 h in steroid-depleted conditions prior to 4 h E_2_ stimulation and RNA extraction. Gene expression data was obtained by hybridising duplicate samples to Illumina HT-12 version 4 BeadChips. Raw microarray data was analysed using GenomeStudio Gene Expression Module (Illumina, San Diego, CA, USA). Differential gene expression analysis was performed using quantile-normalised data incorporating multiple sample testing correction using Benjamini and Hochberg false discovery rate (*p* < 0.05). Gene expression data from the siSCR transfected samples was used as the reference gene expression dataset. Genes up- or down-regulated 1.5 fold in KDM4B-depleted versus siSCR transfected cells were identified by dividing siKDM4B#1 transfected cell gene signal intensities by siSCR transfected cell gene signal intensities. Only genes with a differential *p*-value < 0.05 (as determined by the GenomeStudio Illumina Custom differential expression algorithm) were used in subsequent analyses. Genes were further filtered to exclude genes with an insignificant detection *p*-value (*p* > 0.05) and to exclude genes without a gene symbol/ID and those with only locus specific entries.

For KDM3A and FOXA1 regulated gene signatures, MCF-7 cells were transfected with siSCR, siKDM3A#1, or siFOXA1#1, as described above, and grown for 48 h in steroid-depleted conditions prior to 6 h E_2_ stimulation and RNA extraction. Gene expression data was obtained by hybridising triplicate samples to Illumina HT-12 version 4 BeadChips. Data was analysed as described above. siKDM3A and siFOXA1 regulated genes were identified as genes up- or down- regulated 1.5 fold in siSKDM3A#1/siKDM4B#1 transfected cells compared to E_2_ treated siSCR transfected cells. All microarray data discussed in this publication have been deposited in NCBI’s Gene Expression Omnibus (GEO-https://www.ncbi.nlm.nih.gov/geo/) and are accessible through GEO Series accession numbers GSE135427 and GSE124270.

Genes that were KDM3A/KDM4B/FOXA1 co-regulated were analysed using the Gene Set Enrichment Analysis (GSEA) software (Broad Institute, Cambridge, MA, USA; [67,68]). Identified co-regulated genes were ranked for analysis based on the mean differentiation score calculated by the GenomeStudio software. Ranked lists were compared against the hallmark gene set and GO gene sets from the Molecular Signatures Database v6.2 (MSigDB, Broad Institute). Enriched gene sets with a nominal *p*-value < 0.05 were included in subsequent analyses.

### 4.4. Co-Immunoprecipitation

MCF-7 and T47D cells were grown for 72 h in steroid-depleted or E_2_-stimulated growth conditions in 90 mm dishes. Following incubation, cells were lysed in 1 mL of lysis buffer (50 mM Tris, 150 mM NaCl, 1 mM DTT, 1 mM PMSF, 1% NP40, 1× Protease Inhibitor Cocktail (Roche, Basel, Switzerland)) and subject to immunoprecipitation using 2 µg of KDM3A, KDM4B, or isotype control antibodies (Supplementary Table S7) and Protein G Sepharose beads (GE Healthcare, Chicago, IL, USA). Resultant immunoprecipitates were boiled in SDS sample buffer and subject to western analysis using antibodies specific for KDM3A and KDM4B.

### 4.5. Chromatin Immunoprecipitation (ChIP)

Cells were grown in steroid-depleted medium for 48 h (FOXA1, p300, BRD4, H3K27ac ChIPs) or 72 h (KDM3A and KDM4B ChIPs) and were then treated with vehicle or E_2_ for 45 min. Cells were harvested and subject to ChIP, as previously described [47], using 2 µg of indicated antibodies (Supplementary Table S7). Following ChIP, eluted DNA and input samples underwent cross-link reversal and qPCR analysis using primers specific to estrogen response elements in either the *pS2* promoter, *GREB1*, *CCND1* enhancers, or a *pS2* control region which does not bind ER (Supplementary Table S8 for primer sequences). Data was calculated as % input as previously described [47] and presented as the average fold difference of % input between different experimental arms of at least two independent experiments (detailed in figure legends). 

### 4.6. Cell Growth and Cell Cycle Analysis

MCF-7 and T47D cells were grown in steroid-depleted conditions for 48 h and were then treated with E_2_ for 24 h before transfection with dual knockdown siRNA mixtures. Briefly, cells were transfected with either 25 nM siSCR, 25 nM siRNA mixtures of siSCR with either siKDM3A#2, siKDM3A#3, or siKDM4B#2 (single gene knockdowns—siSCR/si3A#2, siSCR/si3A#3, siSCR/si4B#2), or 25 nM siRNA mixtures of siKDM3A#2 and siKDM4B#2 or siKDM3A#3 and siKDM4B#2 (dual gene knockdowns—si3A#2/si4B#2, si3A#3/si4B#2). Cells were grown for 96 h and either monitored for cell growth by cell counts, by Incucyte Zoom live cell imaging (Essen Bioscience, Ann Arbor, MI, USA), or cell cycle analysis by propidium iodide flow cytometry as previously described [20]. The Incucyte Zoom live cell imager was used to assess relative cell confluence every 6 h between transfected cells, as described in [20]. Cell confluence for each experimental arm was normalised to the 0 h time point (point of transfection) and the relative change in cell confluence calculated for each time point thereafter. Data are presented as the mean relative cell confluence at each time point. Cells counts were performed using a haemocytometer at the end of the experiment. Data were represented as the average fold difference in cell number relative to siSCR transfected cells. 

### 4.7. Development of MCF-7-shRNA Cells

A pTRIPZ-inducible lentiviral KDM3A targeting shRNA vector (Thermo Scientific) was packaged into a virus in HEK293T cells using pMD2.G and pPAX2 packaging vectors. Briefly, 80% confluent cells growing in 150 mm tissue culture dishes were transfected using TransIT-LT1 Transfection Reagent (Geneflow, Lichfield, UK) with 2.25 µg pMD2.G, 6.75 µg pPAX2, and 9 µg pTRIPZ following the manufacturer’s instructions. Twenty-four hours post transfection, tissue culture media was replaced. Over the following 48 h, virus containing media was harvested and viral particles isolated by ultracentrifugation. The virus was re-suspended in 1 mL of RPMI-1640 containing 1 µg/mL puromycin.

MCF-7 cells growing in 30 mm culture dishes were transduced with 100 µL of re-suspended virus and treated with 5 µg/mL of polybrene and 1 µg/mL puromycin. A clonal population of MCF-7-shRNA cells were produced. shRNA expression and KDM3A depletion was confirmed by treating cells for 96 h with vehicle or 1 µg/mL doxycycline and assessing RFP expression by fluorescent microscopy, KDM3A protein levels by western blot, and *KDM3A*, *pS2*, and *GREB1* expression by qPCR. To assess the phenotypic effects of shRNA-induced KDM3A depletion, cells were plated in 1 µg/mL doxycycline-containing media and cell growth analysed by the Incucyte Zoom live cell imager. Cells were also subject to cell counts and propidium iodide cell cycle analysis 96 h post plating.

### 4.8. In Vivo Mouse Study and Immunohistochemistry

All of the in vivo experiments were reviewed and approved by the relevant institutional animal welfare committees (Project License—PPL 60/4222) and performed according to the Guidelines for the Welfare set out by an ad hoc committee of the National Cancer Research Institute [69] and national law. Female athymic CD1 nude mice (Charles Rivers, Ramsgate, Kent, UK) were maintained individually in ventilated cages and handled in lamina flow hoods under specific pathogen free conditions.

Ten CD1 nude mice implanted with 60 day slow release 17β-estradiol (0.18 mg/pellet) pellets (Innovative Research of America) were subcutaneously injected with MCF-7-shRNA cells, 1 × 10^7^ cells in 50 µL per animal, and were suspended in a mixture of matrigel (BD biosciences, San Jose, CA, USA) and RPMI growth media (1:1 *v*:*v*). Engraftments of MCF-7-shRNA cells were successful in six mice and xenografts used in subsequent analyses. Five days following implantation, half the mice were given continuous access to doxycycline supplemented feed (625 ppm doxycycline per 1 kg Diet, Special Diets Services) while the control group were provided non-supplemented feed. Tumours were measured using callipers at least every 4 days and all xenografts were excised 51 days after implantation, or if they had reached a diameter of 1 cm in any direction before that point.

Resultant xenografts were stored in formalin and were paraffin embedded before sectioning. Antigen retrieval was performed by pressure cooking sectioned slides in 10 mM citrate pH 6.0 before immunohistochemistry using anti-KDM3A, -H3K9me2, and -H3K27ac antibodies (Supplementary Table S7) as described [70]. Stained slides were scanned using an Aperio CS2 (Leica Biosystems, Wetzlar, Germany) and analysed using Spectrum TM software (Aperio) to generate Histoscores for each sample. Briefly, stained nuclei were given a score of 0, 1, 2, or 3 based on staining intensity. An overall histoscore was calculated using the equation: (% of nuclei with a score of 1) + 2 × (% of nuclei with score of 2) + 3 × (% of nuclei with score of 3).

## 5. Conclusions

The present study provides evidence of a co-operative role between the histone demethylase enzymes KDM3A and KDM4B in regulating ER-signalling in models of breast cancer. This is the first study to demonstrate a co-operative role between these two enzymes, with data suggesting that the KDM3A/KDM4B co-regulatory axis regulates FOXA1 and subsequent ER deposition on chromatin. Importantly, the finding that dual depletion of KDM3A and KDM4B has a greater anti-proliferative effect than depletion of either enzyme on its own, suggests that dual targeting of these enzymes may be a novel therapeutic approach for both ER-positive and endocrine therapy-resistant disease.

## Supplementary Materials

The following are available online at https://www.mdpi.com/2072-6694/11/8/1122/s1, Figure S1: KDM3A interacts with KDM4B in T47D cells, Figure S2: KDM3A and KDM4B down-regulates the expression of ER target genes, Figure S3: Dual KDM3A- and KDM4B-depletion enhances ER target gene down-regulation, Figure S4: KDM3A-depletion reduces global FOXA1 chromatin association in ER positive BC cells, Figure S5: KDM3A is a key regulator of p300 and BRD4 chromatin function, Figure S6: GSEA of genes down-regulated upon FOXA1 knockdown, Figure S7: Effect of doxycycline induced KDM3A knockdown in MCF-7-shKDM3A cells, Figure S8: In vivo effect of doxycycline induced KDM3A knockdown in MCF-7-shKDM3A mouse xenografts, Figure S9: Inhibition of cell growth is enhanced following KDM3A- and KDM4B-dual knockdown compared to the depletion of either enzyme on its own in MCF-7 cells. Table S1: Gene Set Enrichment analysis of KDM3A/KDM4B/FOXA1 co-regulated genes against the “hallmark” AND “GO” MSigDB gene set collection, Table S2: Gene Set Enrichment analysis of KDM3A/FOXA1 co-regulated genes against the “hallmark” AND “GO” MSigDB gene set collection, Table S3: Gene Set Enrichment analysis of KDM3A/FOXA1 co-regulated genes against the “hallmark” AND “GO” MSigDB gene set collection, Table S4: Gene Set Enrichment analysis of KDM4B/FOXA1 co-regulated genes against the “hallmark” AND “GO” MSigDB gene set collection, Table S5: Gene Set Enrichment analysis of KDM3A/KDM4B co-regulated genes against the “hallmark” AND “GO” MSigDB gene set collection, Table S6: siRNA sequences, Table S7: Antibody details, Table S8: Primer sequences.
