# Supplementary material for: The Histone Demethylase Enzymes KDM3A and KDM4B Co-Operatively Regulate Chromatin Transactions of the Estrogen Receptor in Breast Cancer
Source: Cancers (Basel). 2019 Aug 6;11(8):1122. doi: 10.3390/cancers11081122 (PMC6721541; doi:10.3390/cancers11081122)
Supplement: Supplementary file 1 [file cancers-11-01122-s001.pdf]

Supplementary Materials

The Histone Demethylase Enzymes KDM3A and KDM4B Co-Operatively Regulate Chromatin Transactions of the Estrogen Receptor in Breast Cancer

Dominic Jones, Laura Wilson, Huw Thomas, Luke Gaughan and Mark A. Wade

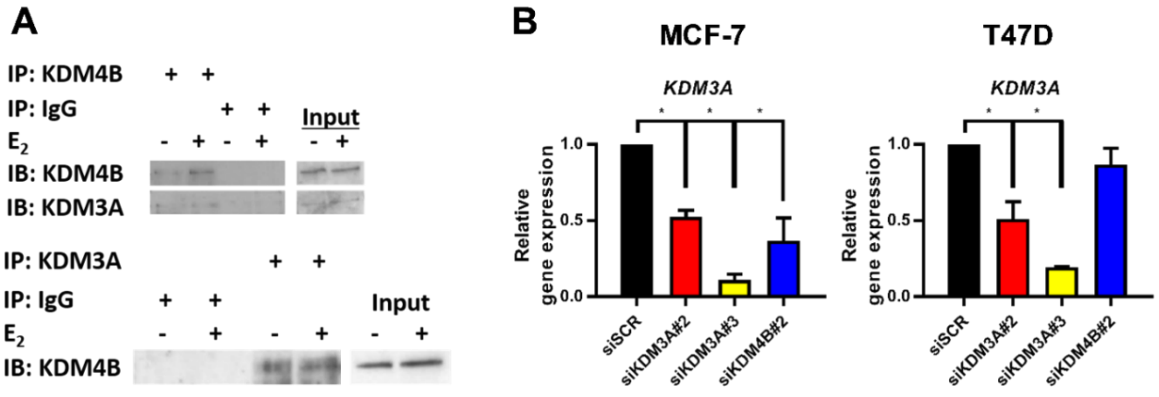

Figure S1. KDM3A interacts with KDM4B in T47D cells. (**A**) T47D cells grown in steroid-depleted ($-E_2$) and 10 nM E_2-supplemented ($+E_2$) media were subject to immunoprecipitation (IP) using anti-KDM4B, anti-KDM3A or isotype control (IgI) antibodies before western blot analysis (IB) using reciprocal antibodies. (**B**) MCF-7 and T47D cells were transiently transfected with either siSCR, siKDM3A#2, siKDM3A#3 or siKDM4B#2 and grown in steroid-depleted conditions for 48 h prior to treatment with E_2 for 4 h and subsequent RNA extraction. Resultant cDNA was analysed for *KDM3A* expression. Data are the average of three independent experiments ± SEM. Gene expression is shown relative to that measured in siSCR transfected cells. *p*-values were determined by Student's *t*-test (*denotes $p < 0.05$).

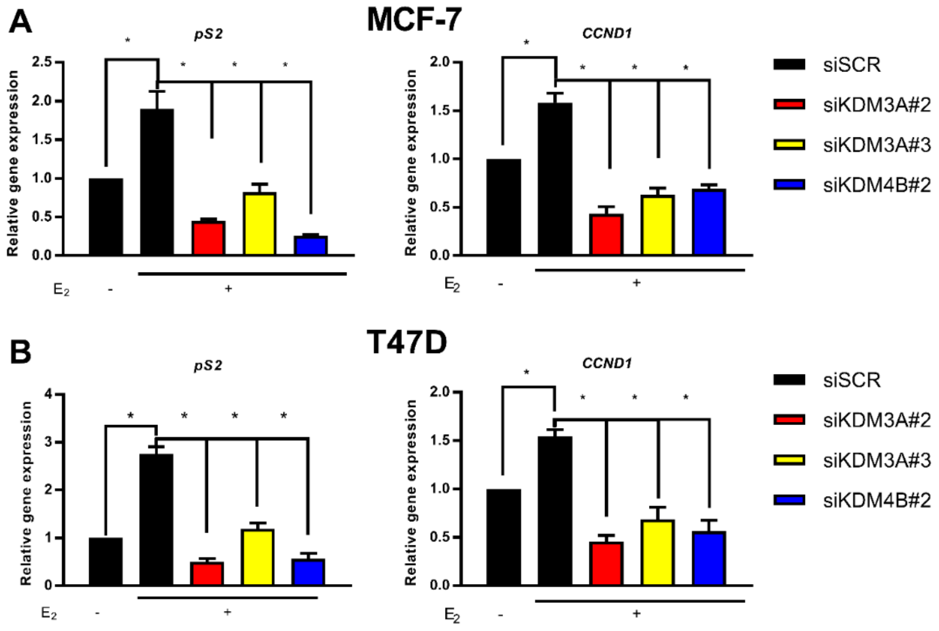

Figure S2. KDM3A and KDM4B down-regulates expression of ER target genes. MCF-7 (**A**) and T47D (**B**) cells were transfected either with siSCR, KDM3A targeting siRNAs (siKDM3A#2 and siKDM3A#3) or an KDM4B targeting siRNA (siKDM4B#2). Cells were grown in steroid-depleted medium for 48 h prior to 4 h treatments with vehicle or 10 nM E_2 prior to RNA extraction. Resultant cDNA was analysed for *pS2* and *CCND1* expression by qPCR. Data are the average of three independent experiments ± SEM. Gene expression is shown relative to that measured in vehicle treated siSCR transfected cells. *P*-values were determined by Turkey's multiple comparisons test (*denotes $p < 0.05$).

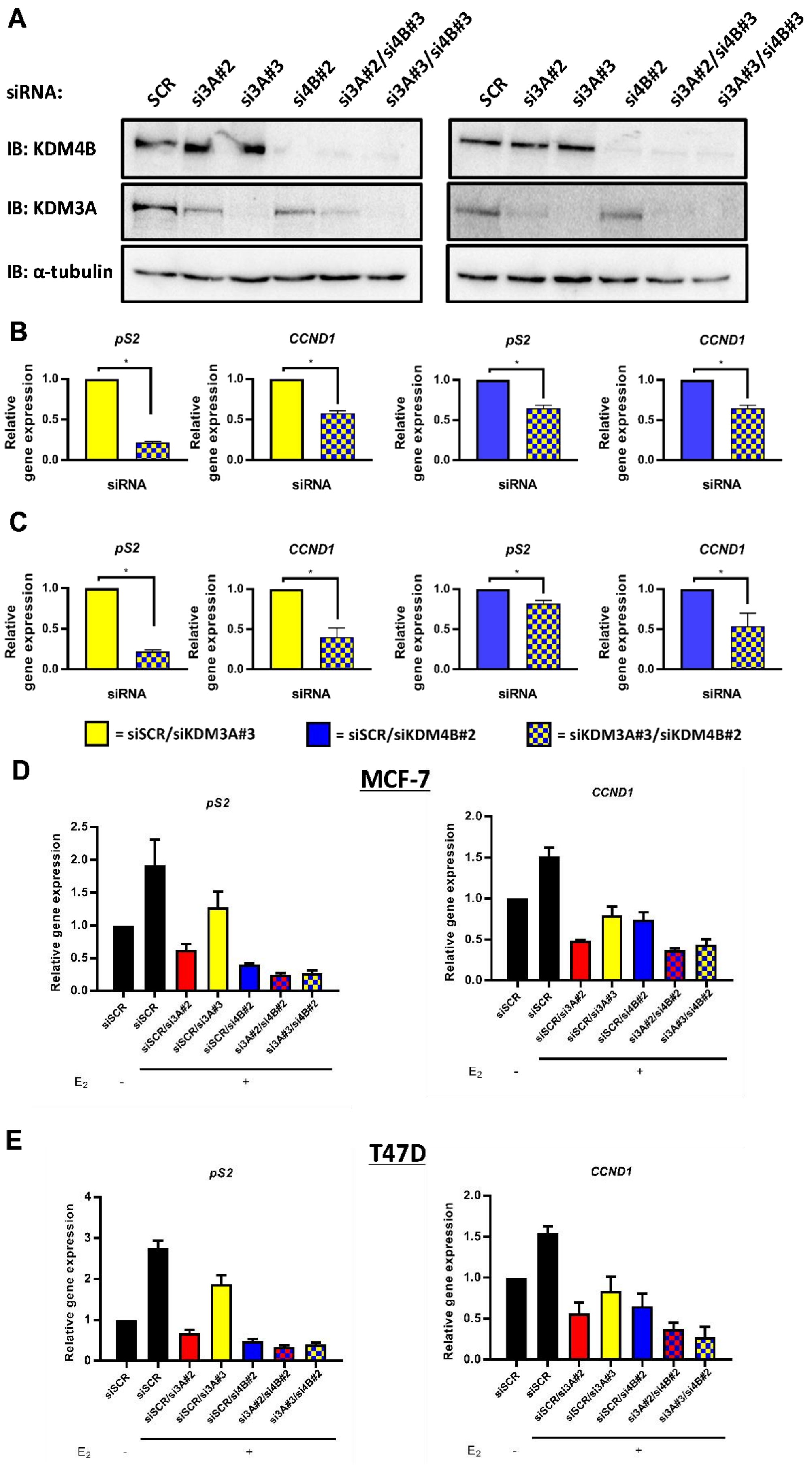

Figure S3. Dual KDM3A- and KDM4B-depletion enhances ER target gene down-regulation. (**A**) MCF-7 or T47D cells were transfected with either 25 nM siSCR, a 25 nM siRNA mixture of siSCR and si3A#2

(si3A#2), siSCR and si3A#3 (si3A#3), siSCR and si4B#2 (si4B#2), si3A#2 and si4B#2 (si3A#2/si4B#2) or si3A#3 and si4B#2 (si3A#3/si4B#2) prior to western blot analysis using antibodies specific to KDM4B, KDM3A and α-tubulin. α-tubulin was used to compare protein loading between samples. (**B** and **C**) *pS2* and *CCND1* gene expression in MCF-7 (**B**) or T47D (**C**) cells transfected with either an siRNA mixture of siSCR and siKDM3A#3 or siSCR and siKDM4B#2 (single knockdowns) and compared with expression in cells transfected with an siRNA cocktail of siKDM3A#3 and siKDM4B#2 (dual knockdown). Cells were transfected and grown in steroid depleted media for 72 h prior to stimulation with E_2 for 6 h and RNA extraction. Data are an average of 3 repeats ± SEM. Data is expressed relative to gene expression in single gene knockdown samples. *P* values were determined by Students T test (*= $p < 0.05$). (**D** and **E**) MCF-7 or T47D cells were transfected with either 25 nM siSCR, a 25 nM siRNA mixture of siSCR and si3A#2 (siSCR/si3A#2), siSCR and si3A#3 (siSCR/si3A#3), siSCR and si4B#2 (siSCR/si4B#2), si3A#2 and si4B#2 (si3A#2/si4B#2) or si3A#3 and si4B#2 (si3A#3/si4B#2) and grown for 72 h in steroid depleted media prior to stimulation with E_2 for 6 h and RNA extraction. RNA was also extracted from siSCR transfected cells which had not undergone E_2 stimulation ($-E_2$). qPCR data are an average of 3 repeats ± SEM and is expressed relative to gene expression in $-E_2$ treated siSCR transfected cells.

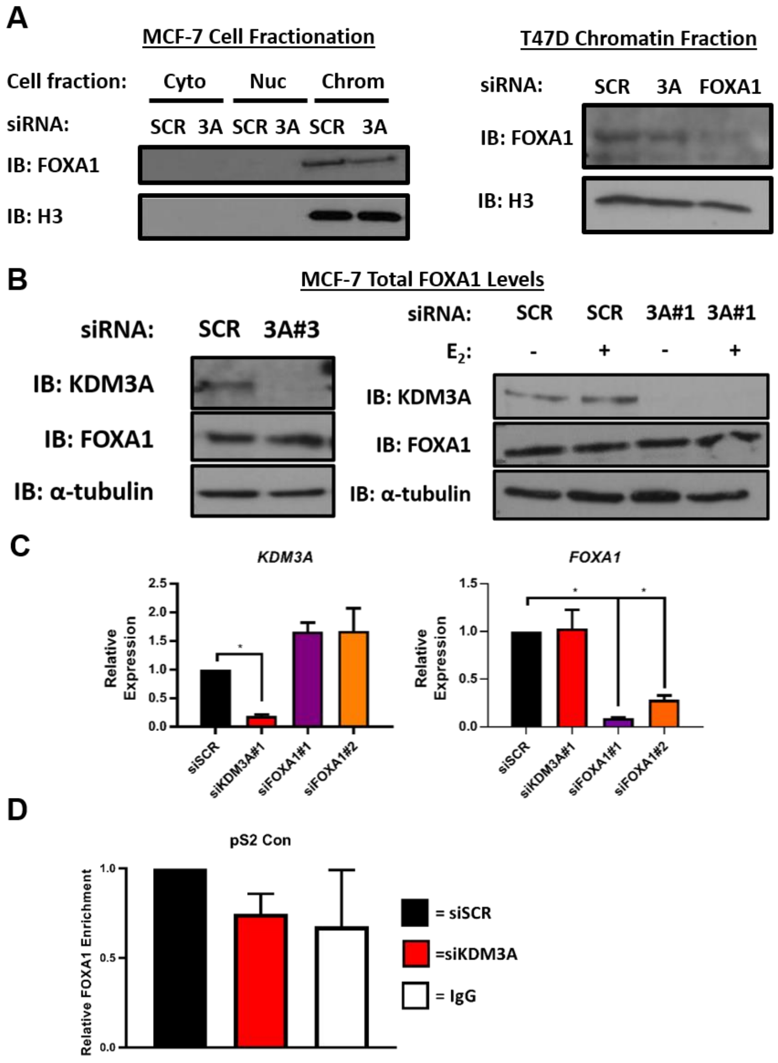

Figure S4. KDM3A-depletion reduces global FOXA1 chromatin association in ER positive BC cells. (**A**) MCF-7 and T47D cells were transiently transfected with either siSCR or siKDM3A#1. Cells were harvested after 48 h then cell fractionation was performed prior to western blot analysis of proteins from each cell fraction (only chromatin fraction for T47D cells) using antibodies specific to FOXA1 and histone H3. H3 was used to compare protein loading between chromatin fractions. (**B**) MCF-7 cells were transiently transfected with either siSCR or siKDM3A#3 (left panel) or siKDM3A#1 (right panel) and grown for 48 h prior to western blot analysis using antibodies specific to KDM3A, FOXA1

and α-tubulin. Cells in the right panel were grown in steroid depleted ($-E_2$) or 10 nM E_2 supplemented conditions. (**C**) MCF-7 cells were transiently transfected with either a non-silencing control siRNA (siSCR), a KDM3A targeting siRNA (siKDM3A#1) or FOXA1 targeting siRNAs (siFOXA1#1 and siFOXA1#2) and grown in steroid-depleted medium for 48 h prior to 6 h treatments with 10 nM E_2. RNA was extracted and resultant cDNA was analysed for *KDM3A* and *FOXA1* expression by qPCR. Data are the average of three independent experiments ± SEM. Gene expression is shown relative to that measured in siSCR transfected cells. *P*-values were determined by Student's *t*-test (*denotes $p < 0.05$). (**D**) MCF-7 cells were transiently transfected with either siSCR or siKDM3A#1 and grown in steroid-depleted conditions for 48 h prior to treatment with 10 nM E_2 for 45 min followed by ChIP analysis using antibodies specific to FOXA1 and an isotype control (IgG). Enrichment of FOXA1 at a region within the *pS2* promoter which the ER does not bind (pS2 Con) was assessed by qPCR. Data are an average of 2 independent experiments ± SEM and is expressed relative to the level of enrichment measured in siSCR transfected cells.

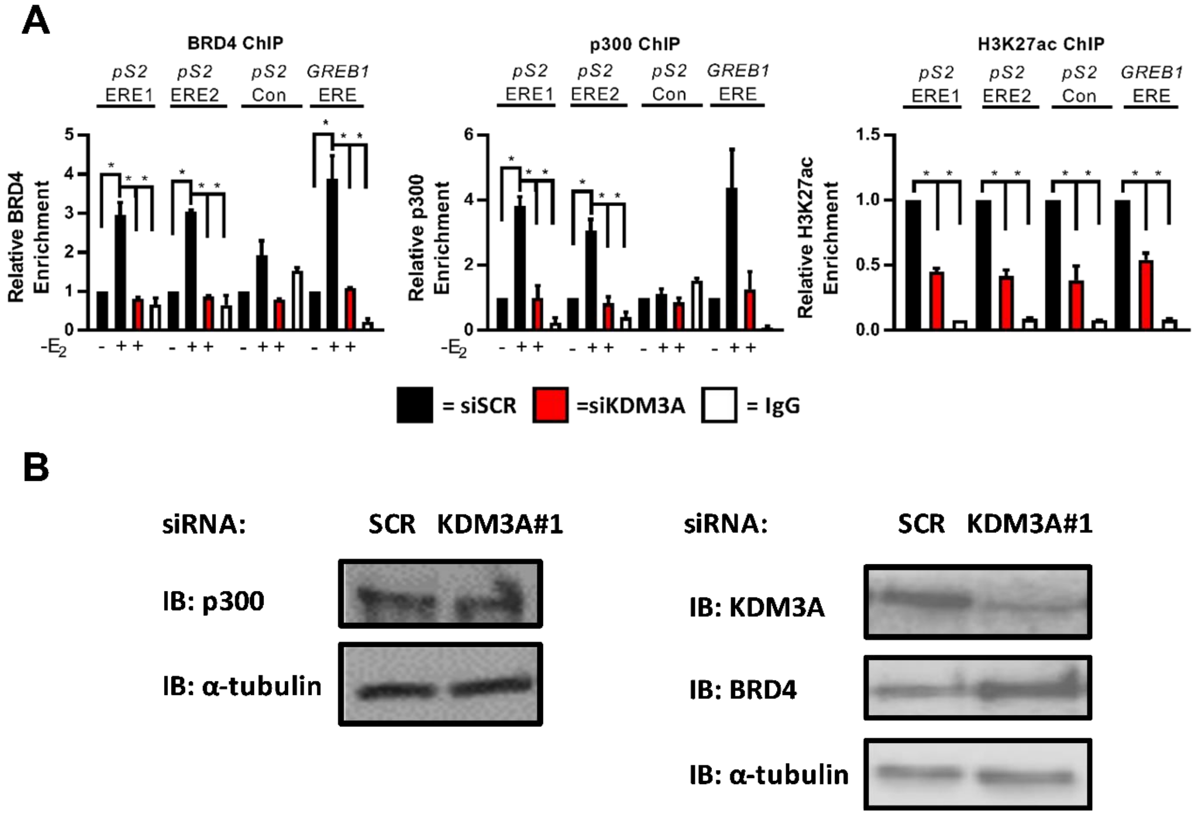

Figure S5. KDM3A is a key regulator of p300 and BRD4 chromatin function. (**A**) MCF-7 cells were transiently transfected with either siSCR or siKDM3A#1 and grown in steroid-depleted conditions for 48 h prior to treatment with vehicle or 10 nM E_2 for 45 min followed by ChIP analysis using antibodies specific to BRD4, p300, H3K27ac or isotype controls (IgG). Enrichment of BRD4, p300 and H3K27ac at *pS2* and *GREB1* EREs as well as a region within the *pS2* promoter which the ER does not bind (pS2 Con) was assessed by qPCR. Data are an average of 2 independent experiments ± SEM and is expressed relative to the level of enrichment measured in vehicle treated siSCR transfected cells. *P*-values were determined by Dunnet's multiple comparisons test (* denotes $p < 0.05$). For H3K27ac ChIP analysis the level of enrichment is expressed relative to E_2-treated siSCR transfected cells. (**B**) MCF-7 cells were transiently transfected with either siSCR or siKDM3A#1 and grown for 72 h prior to western blot analysis using antibodies specific to P300, KDM3A, BRD4 and α-tubulin. α-tubulin was used to compare protein loading between samples.

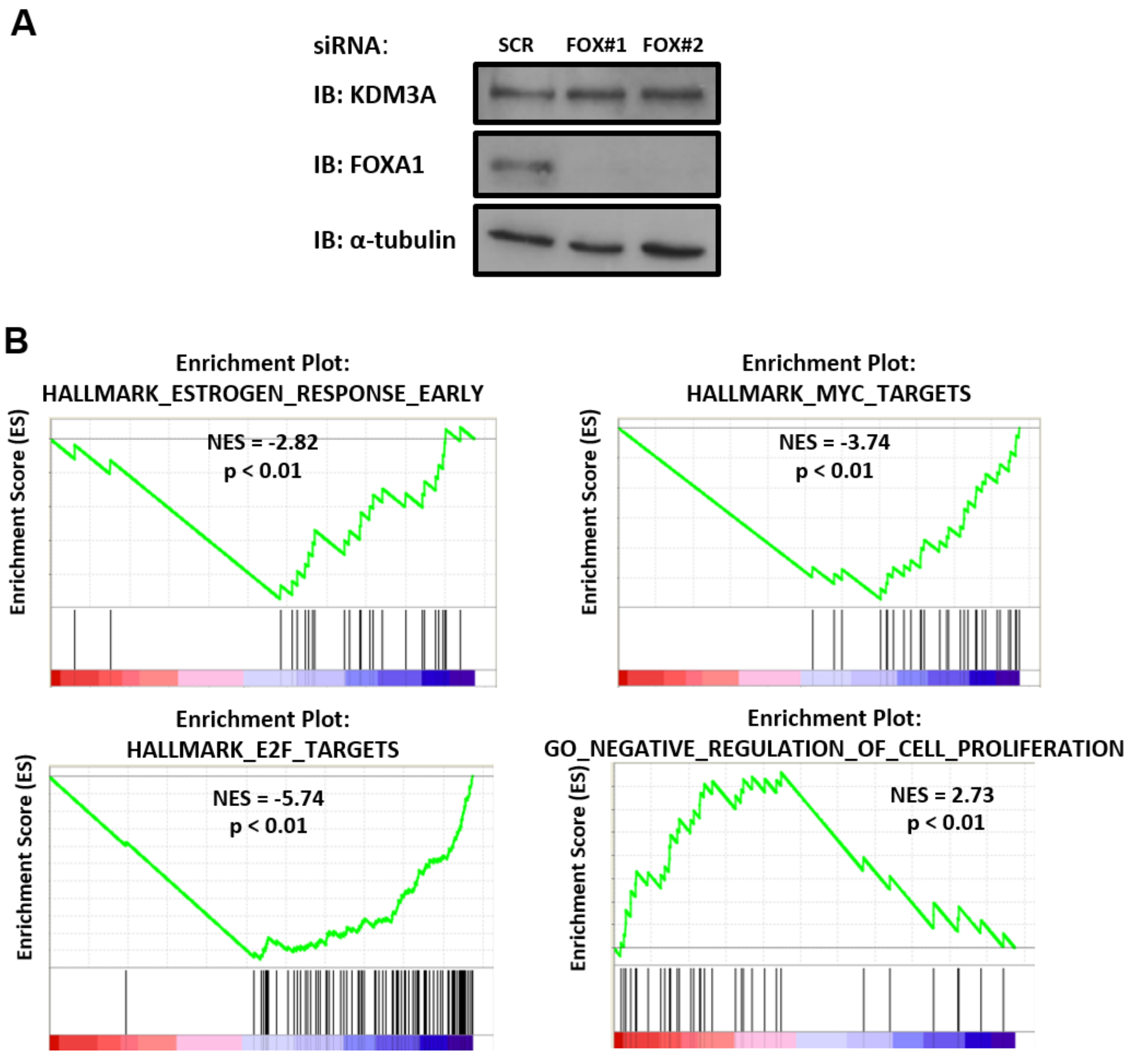

Figure S6. GSEA of genes down-regulated upon FOXA1 knockdown. (**A**) MCF-7 cells were transiently transfected with either siSCR, siFOXA1#1 or siFOXA1#2 and grown for 72 h prior to western blot analysis using antibodies specific to KDM3A, FOXA1 and α-tubulin. α-tubulin was used to compare protein loading between samples. (**B**) GSEA of KDM3A/FOXA1 co-regulated genes demonstrates significant negative enrichment of genes associated with the Hallmark Estrogen Response (early), MYC Target, and E2F Target gene sets and positive enrichment of genes associated with the GO Negative Regulation of Cell Proliferation gene set. NES = Normalised Enrichment Score. p = nominal *p*-value.

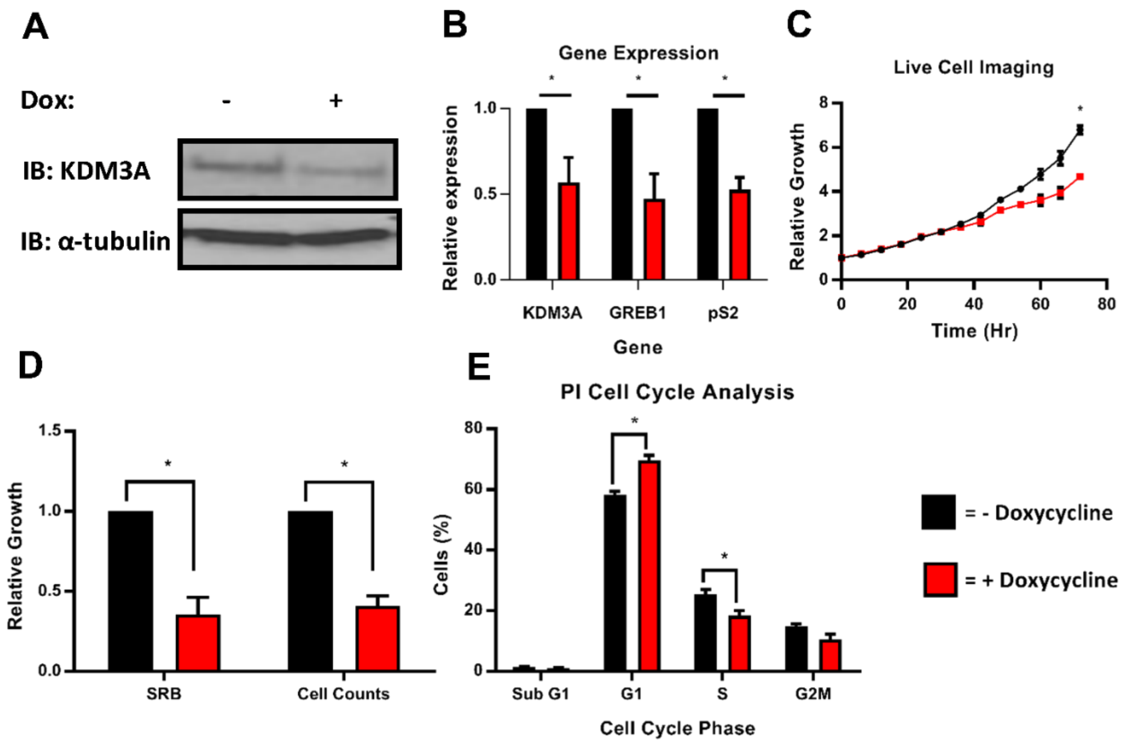

Figure S7. Effect of doxycycline induced KDM3A knockdown in MCF-7-shKDM3A cells. (**A**) MCF-7-shKDM3A cells were treated with vehicle or 1 μg/ml doxycycline (dox) for 72 h prior to western blot analysis using antibodies specific to KDM3A and α-tubulin. α-tubulin was used to compare protein loading between samples. (**B**) MCF-7-shKDM3A cells were treated as in (**A**) prior to RNA extraction. Resultant cDNA was analysed for *KDM3A, pS2* and *GREB1* expression by qPCR. Data are the average of three independent experiments ± SEM. Gene expression is shown relative to that measured in vehicle treated cells. p-values were determined by Student's t-test (*denotes $p < 0.05$). (**C** and **D**) MCF-7-shKDM3A cells were treated as in (**A**). Cell confluence was measured every 6 h by the Incucyte Zoom live cell imager and cells were counted at the end of the experiment. For live cell imaging, data were normalised for each sample to the cell confluence measured at 0 h. For both growth measurements data are the average of three independent experiments ± SEM. p-values of the differences in cell confluence at the final timepoint were determined by Student's t-test (*denotes $p <$ 0.05). (**E**) MCF-7-shKDM3A cells were treated as in (**A**) prior to harvesting for cell cycle analysis by propidium iodide flow cytometry. Data shows the % of cells in each phase of the cell cycle and is the average of three independent experiments ± SEM. P-values were determined by Student's t-test (*denotes $p < 0.05$). These data all mirror gene expression and phenotypic patterns observed in MCF-7 cells transfected with KDM3A targeting siRNA [20].

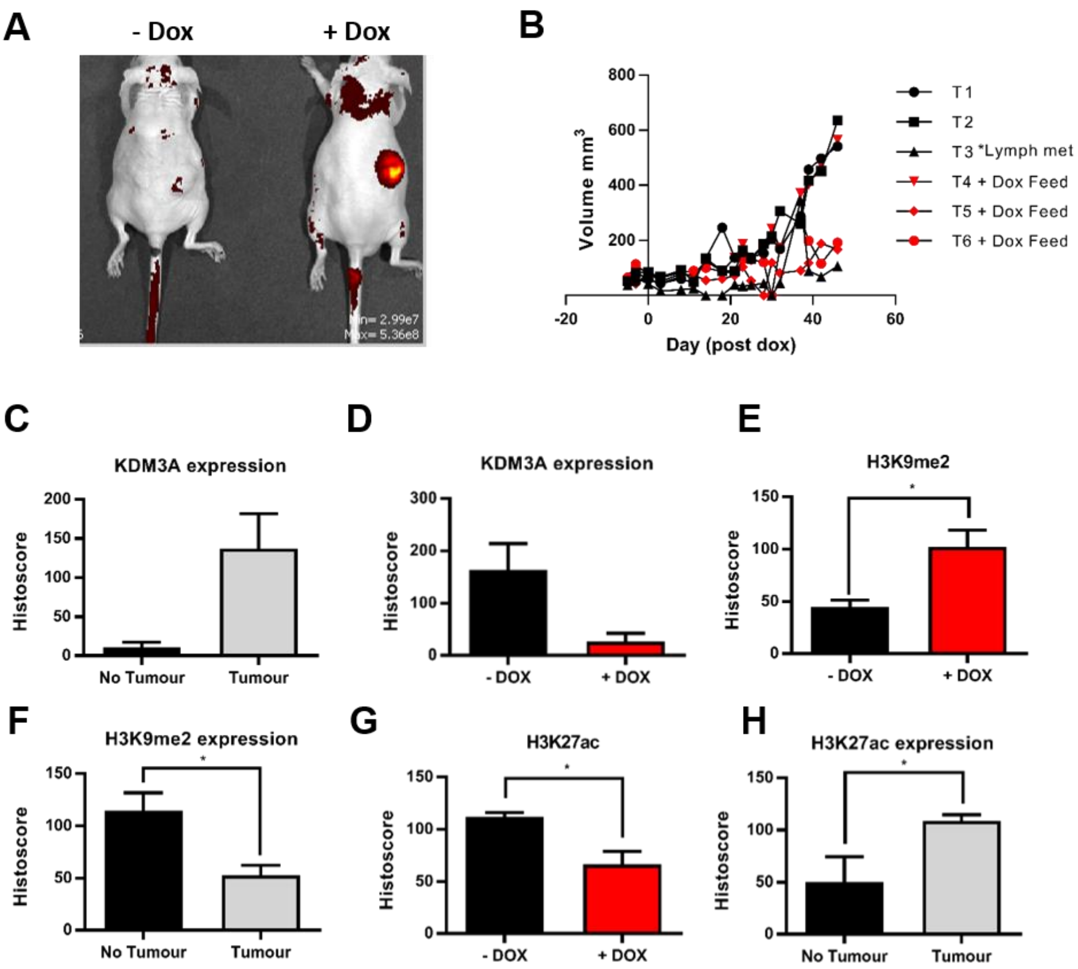

Figure S8. In vivo effect of doxycycline induced KDM3A knockdown in MCF-7-shKDM3A mouse xenografts. MCF-7-shKDM3A cells were subcutaneously injected into 6 immunocompromised mice. Three mice were continually fed doxycycline (+dox) supplemented feed 5 days post-injection and three were not (−dox). (**A**) Doxycycline induced expression of RFP in the mice was established by live fluorescent imaging of RFP signal in both cohorts of mice. (**B**) Growth of the xenografts was monitored over 51 days before all xenografts were excised. T1-3 xenografts were from –dox mice and T4-6 xenografts were from +dox mice. Mouse T3 also had a large lymph node metastases. Immunohistochemistry was performed on excised xenografts using antibodies specific for KDM3A (**C** and **D**), H3K9me2 (**E** and **F**) and H3K27ac (**G** and **H**). The expression of KDM3A and level of respective histone modifications was quantified by derivation of a histoscore using automated scanning software. Data are the average of the histoscores in -dox vs +dox sample cohorts ± SEM. *P*-values were determined by Student's *t*-test (*denotes $p < 0.05$).

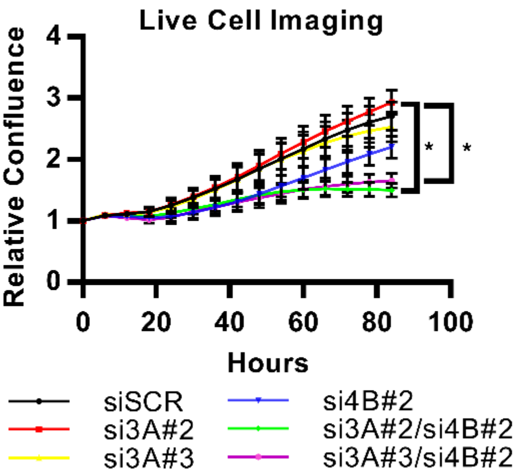

Figure S9. Inhibition of cell growth is enhanced following KDM3A- and KDM4B-dual knockdown compared to depletion of either enzyme on its own in MCF-7 cells. MCF-7 cells were grown in steroid-depleted conditions for 48 h then treated with 10 nM E_2 for 24 h before transfection with either siSCR or single and dual-knockdown siRNA mixtures (see materials and methods) and grown for a further 96 h. Data labels indicate which KDM siRNAs were included in each siRNA mixture. Cell confluence was measured every 6 h post-transfection by the Incucyte Zoom live cell imager. Data was normalised for each experimental arm to the cell confluence measured at 0 h. Data are the average of three independent experiments ± SEM.

Table S1. Gene Set Enrichment analysis of KDM3A/KDM4B/FOXA1 co-regulated genes against the "hallmark" AND "GO" MSigDB gene set collection. Gene sets significantly enriched at nominal *p* value < 0.05 shown. ES = Enrichment Score; NES = Normalised Enrichment Score; NOM = Nominal *p*-value; FDR = False Discovery Rate.

Gene Set Name	Genes	SIZE	ES	NES	NOM p-val	FDR q-val
HALLMARK_G2M_CHECKPOINT	*UCK2, SMC2, DR1, E2F4, KIF11, H2AFX, SETD8, MCM5, CCNF, CCND1, TROAP, CHEK1, AURKA, MCM2, CCNA2*	15	-0.67172	-3.07958	0	0
GO_MITOTIC_CELL_CYCLE	*BECN1, XPC, ENSA, ZW10, ZWILCH, SKP2, CASP2, SMC2, DSCC!, E2F4, KIF11, SETD8, E2F7, MCM5, CCNF, FOXM1, CCDC99, CCND1, CHEK1, AURKA, MCM2, CCNA1*	22	-0.4098	-2.21986	0	0.08001681
GO_CELL_CYCLE	*RRAGD, RAB11FIP3, BECN1, XPC, ENSA, ZW10, GSK3B, ZWILCH, SKP2, CASP2, SMC2, UBE2L3, DSCC1, E2F4, KIF11, H2AFX, SETD8, E2F7, MCM5, CCNF, FOXM1, CCDC99, CCND1, CHEK1, AURKA, MCM2, TRIP13, HJURP, CCNA2*	29	-0.36001	-2.19917	0.00203666	0.045617547
GO_REPRODUCTION	*FOX, ASCL2, ZW10, ERCC1, INHBB, CASP2, SMC2, WDR77, MAMLD1, H2AFX, HEY2, E2F7, CCNF, FZD4, CCND1, SERPINA5, AURKA, STC2, TRIP13*	19	-0.42865	-2.14511	0	0.044176538
GO_CELL_CYCLE_PROCESS	*RRAGD, RAB11FIP3, BECN1, XPC, ENSA, ZW10, GSK3B, ZWILCH, SKP2, CASP2, SMC2, UBE2L3, DSCC1, E2F4, KIF11, H2AFX, SETD8, E2F7, MCM5, CCNF, FOXM1, CCDC99, CCND1, CHEK1, AURKA, MCM2, TRIP13, CCNA2*	28	-0.34672	-2.09957	0.00412371	0.044456087
GO_TISSUE_DEVELOPMENT	*TUFT1, FOS, ASCL2, GSK3B, WDR77, E2F4, RET, ALDH3A2, RPS7, TGM2, HEY2, E2F7, TGFBI, CCND1, STC2*	15	-0.4303	-1.9805	0.00761905	0.07596908
GO_SYSTEM_PROCESS	*MYL5, FOS, FAM107B, NR2F6, PNKD, NAV2, E2F4, SERPINA3, EIF4EBP2, HEY2, TGFBI, FZD4, GPD1L, DTNA, TFF1*	15	-0.38586	-1.72517	0.02574257	0.25421453

Table S2. Gene Set Enrichment analysis of KDM3A/FOXA1 co-regulated genes against the "hallmark" AND "GO" MSigDB gene set collection. Gene sets significantly negatively enriched at nominal *p* value < 0.05 shown. For GO gene sets the top 10 most enriched gene sets (as determined by NES) are shown. ES = Enrichment Score; NES = Normalised Enrichment Score; NOM = Nominal p-value; FDR = False Discovery Rate.

**Gene Set Name**	**SIZE**	**ES**	**NES**	**NOM p-val**	**FDR q-val**
GO_CELL_CYCLE	210	-0.40589	-6.22044	0	0
GO_MITOTIC_CELL_CYCLE	155	-0.45558	-6.18232	0	0
GO_CELL_CYCLE_PROCESS	191	-0.41133	-5.96126	0	0
HALLMARK_G2M_CHECKPOINT	82	-0.55735	-5.75542	0	0
HALLMARK_E2F_TARGETS	94	-0.52409	-5.74381	0	0
GO_ORGANELLE_FISSION	105	-0.4325	-4.96118	0	0
GO_MITOTIC_NUCLEAR_DIVISION	93	-0.43159	-4.73609	0	0
GO_CELL_DIVISION	101	-0.41586	-4.71791	0	0
GO_CELL_CYCLE_PHASE_TRANSITION	56	-0.54173	-4.59743	0	0
GO_CHROMOSOME_SEGREGATION	75	-0.44633	-4.38124	0	0
GO_NUCLEAR_CHROMOSOME_SEGREGATION	68	-0.4673	-4.35632	0	0
GO_SISTER_CHROMATID_SEGREGATION	63	-0.46127	-4.26081	0	0
HALLMARK_MYC_TARGETS_V1	30	-0.56986	-3.74148	0	0
HALLMARK_ESTROGEN_RESPONSE_LATE	35	-0.49162	-3.42071	0	0
HALLMARK_ESTROGEN_RESPONSE_EARLY	25	-0.47238	-2.82168	0	0
HALLMARK_MITOTIC_SPINDLE	42	-0.34099	-2.55283	0	0
HALLMARK_SPERMATOGENESIS	18	-0.41853	-2.10866	0	0.004427
HALLMARK_UV_RESPONSE_UP	25	-0.30857	-1.85163	0.009709	0.018

Table S3. Gene Set Enrichment analysis of KDM3A/FOXA1 co-regulated genes against the "hallmark" AND "GO" MSigDB gene set collection. Gene sets significantly positively enriched at nominal *p* value < 0.05 shown. For GO gene sets the top 10 most enriched gene sets (as determined by NES) are shown. ES = Enrichment Score; NES = Normalised Enrichment Score; NOM = Nominal *p*-value; FDR = False Discovery Rate.

Gene Set Name	SIZE	ES	NES	NOM p-val	FDR q-val
GO_VESICLE_MEDIATED_TRANSPORT	76	0.300248	3.033636	0	0.001859
GO_NEGATIVE_REGULATION_OF_MULTICELLULAR_ORGANISMAL_PROCESS	45	0.368932	2.896784	0	0.001408
GO_NEGATIVE_REGULATION_OF_CELL_PROLIFERATION	37	0.37916	2.7356	0	0.002518
GO_VACUOLE	63	0.280915	2.532955	0	0.010141
GO_REGULATION_OF_MULTICELLULAR_ORGANISMAL_DEVELOPMENT	88	0.226582	2.461576	0.002	0.015405
GO_RESPONSE_TO_EXTERNAL_STIMULUS	96	0.221761	2.438805	0	0.015923
GO_NEGATIVE_REGULATION_OF_DEVELOPMENTAL_PROCESS	46	0.310242	2.434503	0	0.013648
GO_ENDOPLASMIC_RETICULUM	87	0.232026	2.428188	0	0.01263
GO_ANCHORING_JUNCTION	33	0.351335	2.398543	0	0.014081
GO_VACUOLAR_PART	33	0.351335	2.376147	0	0.015462
HALLMARK_ADIPOGENESIS	16	0.448595	2.182417	0	0.009175
HALLMARK_P53_PATHWAY	25	0.350476	2.119951	0	0.005437
HALLMARK_TNFA_SIGNALING_VIA_NFKB	17	0.400589	1.980979	0.0019305	0.008093
HALLMARK_HYPOXIA	22	0.33152	1.817693	0.01377953	0.016547

Table S4. Gene Set Enrichment analysis of KDM4B/FOXA1 co-regulated genes against the "hallmark" AND "GO" MSigDB gene set collection. Gene sets significantly negatively enriched at nominal *p* value < 0.05 shown. For GO gene sets the top 10 enriched gene sets (as determined by NES) are shown. ES = Enrichment Score; NES = Normalised Enrichment Score; NOM = Nominal p-value; FDR = False Discovery Rate.

Gene Set Name	SIZE	ES	NES	NOM p-val	FDR q-val
HALLMARK_G2M_CHECKPOINT	16	-0.62453	-2.99336	0	0
GO_CELL_CYCLE	32	-0.3705	-2.36587	0	0.02949711
GO_REGULATION_OF_CELL_CYCLE_PROCESS	17	-0.47414	-2.33114	0	0.01936348
GO_MITOTIC_CELL_CYCLE	24	-0.40504	-2.28381	0	0.01964054
GO_CELL_CYCLE_PROCESS	31	-0.35317	-2.19918	0	0.03041334
GO_REGULATION_OF_MITOTIC_CELL_CYCLE	15	-0.43745	-2.00923	0	0.07866398
GO_RIBONUCLEOPROTEIN_COMPLEX	16	-0.42904	-1.99955	0.01209677	0.07013489
HALLMARK_ESTROGEN_RESPONSE_EARLY	15	-0.43596	-1.9488	0.01174168	0.00967742
GO_POSITIVE_REGULATION_OF_BIOSYNTHETIC_PROCESS	24	-0.34399	-1.9307	0.01190476	0.09721144
GO_PROTEIN_MODIFICATION_BY_SMALL_PROTEIN_CONJUGATION_OR_REMOVAL	17	-0.38357	-1.8794	0.00590551	0.11299361
GO_SYSTEM_PROCESS	17	-0.38513	-1.84961	0.008	0.11847371
GO_REGULATION_OF_CELL_CYCLE	21	-0.32403	-1.74728	0.01818182	0.18984705

Table S5. Gene Set Enrichment analysis of KDM3A/KDM4B co-regulated genes against the "hallmark" AND "GO" MSigDB gene set collection. Gene sets significantly negatively enriched at nominal *p* value < 0.05 shown. For GO gene sets the top 10 enriched gene sets (as determined by NES) are shown. Genes are detailed for G2M, estrogen response and mitotic cell cycle gene sets. ES = Enrichment Score; NES = Normalised Enrichment Score; NOM = Nominal p-value; FDR = False Discovery Rate.

Gene Set Name	Genes	SIZE	ES	NES	NOM p-val	FDR q-val
HALLMARK_G2M_CHECKPOINT	*HMGA1, SMC2, SLC7A5, MCM5, UCK2, KIF11, CCNF, SETD8, E2F4, DR1, H2AFX, MYC, TROAP, CHEK1, AURKA, MCM2, CCND1, CCNA2*	18	-0.6362683	-3.1116123	0	0
HALLMARK_ESTROGEN_RESPONSE_EARLY	*FOS, CLIC3, SLC37A1, SCARB1, ENDOD1, RASGRP1, INHBB, SLC7A5, CA12, NAV2, RET, FLNB, MSMB, ASB13, TFF1, MYC, TGM2, MICB, STC2, KRT13, CCND1, TSKU, DHRS2*	24	-0.4967949	-2.761527	0	0
HALLMARK_ESTROGEN_RESPONSE_LATE	*FOS, PERP, CLIC3, UNC13B, SCARB1, SLC22A5, SLC7A5, CA12, RET, FLNB, ALDH3A2, SERPINA3, TFF1, UGDH, MICB, KRT13, CCND1, SERPINA5, DHRS2*	19	-0.4150755	-2.1521087	0	0.001909126
GO_REGULATION_OF_PROTEIN_MODIFICATION_PROCESS		25	-0.3530546	-2.0735686	0.00591716	0.44783527
GO_PHOSPHORYLATION		19	-0.4047817	-2.0504553	0	0.25445855
GO_EXTRACELLULAR_SPACE		22	-0.3830342	-2.0491536	0.004106776	0.17217496
GO_MITOTIC_CELL_CYCLE	*ENSA, ZW10, BBS4, BECN1, XPC, CASP2, ZWILCH, SKP2, SMC2, BLM, DSCC1, MCM5, KIF11, CCNF, SETD8, E2F4, FOXM1, CCDC99, E2F7, CHEK1, AURKA, MCM2, CCND1, CCNA2*	24	-0.3557692	-2.003129	0	0.16170464
GO_POSITIVE_REGULATION_OF_BIOSYNTHETIC_PROCESS		26	-0.3367246	-1.9456638	0.004210526	0.1849985
GO_EPITHELIUM_DEVELOPMENT		19	-0.3782168	-1.9213741	0.016260162	0.1805515
GO_CELL_CYCLE		39	-0.2600362	-1.8664509	0.015748031	0.21324752
GO_ENZYME_BINDING		41	-0.2529971	-1.811416	0.018	0.25635016
GO_PHOSPHATE_CONTAINING_COMPOUND_METABOLIC_PROCESS		33	-0.279928	-1.7792653	0.012320329	0.27072427
GO_ADENYL_NUCLEOTIDE_BINDING		26	-0.2928039	-1.736169	0.015936255	0.30771494

Table S6. siRNA sequences.

siRNA Name	Sequence (5'-3')
siSCR	UUCUCCGAACGUGUCACGU
siKDM3A#1	CAAACUGCCCUUGUUCAAA
siKDM3A#2	GAGAUACUGCUUGGCUGUA
siKDM3A#3	GCACAGUCCUCCAUACGUU
siKDM4B#1	GGCAUAAGAUGACCCUCAU
siKDM4B#2	CAAAUACGUGGCCUACAUA
siFOXA1#1	GAGAGAAAAAAUCAACAGC
siFOXA1#2	GCACUGCAAUACUCGCCUU

Table S7. Antibody details.

	Use		
**Antibody**	**Western Analysis**	**Immunoprecipitation**	**Immunohistochemistry**
KDM3A A301-539A - Bethyl laboratories		X	
KDM3A 12835-1-AP - ProteinTech	X		X
KDM4B A301-478A - Bethyl laboratories		X	
FOXA1 C15410231 - Diagenode	X	X	
H3K27ac A7071-001P - Diagenode		X	X
H3K9me2 C15410060 - Diagenode			X
BRD4 A301-085A - Bethyl laboratories	X	X	
p300 (sc-585) Santa Cruz Biotechnology	X	X	
α-tubulin T9026 - Sigma	X		
Histone H3 ab1791 - Abcam	X		
Rabbit IgG - Diagenode		X	
**Secondary Antibodies**			
Polyclonal Swine Anti-Rabbit HRP - DAKO	X		
Polyclonal Rabbit Anti-Mouse HRP - DAKO	X		
Anti-Mouse IgG MP-7402 – Vector Labs			X
Anti-Rabbit IgG MP-7401 – Vector Labs			X

Table S8. Primer sequences.

mRNA Primers	Sequence 5'-3'
RPL13A F	CCTGGAGGAGAAGAGGAAAGAGA
RPL13A R	TTGAGGACCTCTGTGTATTTGTCAA
KDM3A F	GGAGCTCCACATCAGGTTCATAA
KDM3A R	TTCAGCCACTTTGATGCAGC
pS2 F	GTGTCACGCCCTCCCAGT
pS2 R	GGACCCCACGAACGGTG
CCND1 F	ACTACCGCCTCACACGCTTC
CCND1 R	AGTCCGGGTCACACTTGATCA
FOXA1 F	GGGGGTTTGTCTGGCATAGC
FOXA1 R	GCACTGGGGGAAAGGTTGTG
GREB1 F	CAAAGAATAACCTGTTGGCCCTGC
GREB1 R	GACATGCCTGCGCTCTCATACTTA
**ChIP Primers**	**Sequence 5'-3'**
pS2 ERE1 F	TTCCGGCCATCTCTCACTAT
pS2 ERE1 R	ATGGGAGTCTCCTCCAACCT
pS2 ERE2 F	CCATGGGAAAGAGGGACTTT
pS2 ERE2 R	TGGTCAAGCTACATGGAAGG
pS2 Control F	AATACCTGAGGACCCCAACC
pS2 Control R	TCTTCACTCTCCTCGCATTG
GREB1 ERE F	AGCAGTGAAAAAAAGTGTGGCAACTGGG
GREB1 ERE R	CGACCCACAGAAATGAAAAGGCAGCAAACT
CCND1 ERE F	CAGTTTGTCTTCCCGGGTTA
CCND1 ERE R	TCATCCAGAGCAAACAGCAG

© 2019 by the authors. Licensee MDPI, Basel, Switzerland. This article is an open access article distributed under the terms and conditions of the Creative Commons Attribution (CC BY) license (http://creativecommons.org/licenses/by/4.0/).
